# Ecosystem retrogression enhances cross‐domain microbial stability and increases the genetic potential for nutrient cycling

**DOI:** 10.1002/mlf2.70087

**Published:** 2026-07-19

**Authors:** Javier A. Ceja‐Navarro, Dishant Patel, Garret Genco, Alyssa Byer, Daliang Ning, Kenneth H. Wan, Susan E. Celniker, Jizhong Zhou, Paul Dijkstra, Bruce A. Hungate, Jennifer Pett‐Ridge, Eoin L. Brodie

**Affiliations:** ^1^ Center for Ecosystem Science and Society Northern Arizona University Flagstaff Arizona USA; ^2^ Department of Biological Sciences Northern Arizona University Flagstaff Arizona USA; ^3^ Bioengineering and Biomedical Sciences Department, Biological Systems and Engineering Division Lawrence Berkeley National Laboratory Berkeley California USA; ^4^ Institute for Biodiversity Science and Sustainability California Academy of Sciences San Francisco California USA; ^5^ Department of Environmental Science, Policy and Management University of California Berkeley Berkeley California USA; ^6^ Institute for Environmental Genomics University of Oklahoma Norman Oklahoma USA; ^7^ School of Biological Sciences University of Oklahoma Norman Oklahoma USA; ^8^ School of Civil Engineering and Environmental Sciences University of Oklahoma Norman Oklahoma USA; ^9^ School of Computer Science University of Oklahoma Norman Oklahoma USA; ^10^ Physical and Life Sciences Directorate Lawrence Livermore National Laboratory Livermore California USA; ^11^ Life & Environmental Sciences Department University of California Merced Merced California USA; ^12^ Innovative Genomics Institute University of California Berkeley Berkeley USA; ^13^ Ecology Department, Earth and Environmental Sciences Lawrence Berkeley National Laboratory Berkeley California USA

**Keywords:** cross‐domain microbiome, ecosystem retrogression, nutrient limitation, soil chronosequence

## Abstract

Ecosystem retrogression drives nutrient depletion, reduced productivity, and profound reorganization of soil microbial communities. Using amplicon sequencing and genome‐resolved metagenomics, we examined how cross‐domain microbial networks and functional gene potential respond to long‐term phosphorus and nitrogen limitation along the well‐characterized Ecological Staircase chronosequence in Mendocino, California, USA. Microbial diversity and abundance declined sharply with terrace age for prokaryotes, predatory protists, and bacteriophages, whereas fungi and phototrophic protists increased in nutrient‐depleted, acidic soils. These compositional shifts were accompanied by major changes in reconstructed microbial networks: relative modularity increased alongside robustness, indicating adaptive reorganization that may sustain ecosystem function under resource scarcity. Fungi emerged as central stabilizers in these restructured networks, carrying enriched genetic potential to degrade plant polymers and mobilize phosphorus and nitrogen. Despite a decline in overall phage diversity, the relative abundance of phages encoding phosphorus‐mobilizing auxiliary metabolic genes increased, suggesting that viral contributions to host phosphorus metabolism may be enhanced under nutrient limitation. Together, these results demonstrate that ecosystem retrogression drives cross‐domain microbial reorganization toward fewer but more interconnected lineages, characterized by greater integration of functional genetic potential. This reorganization enhances the potential for functional resilience under extreme nutrient limitation, revealing how microbial networks adapt to maintain the capacity for nutrient cycling and stability as soils age and fertility declines.

## Impact statement

As ecosystems age and lose nutrients, most microbes struggle, but some reorganize to keep life going. This study shows that bacteria, fungi, protists, and viruses rewire their interactions to maintain the potential for ecosystem function even in severely nutrient‐depleted soils. Using DNA sequencing and network analysis, we reveal how microbes adapt to harsh conditions and support one another. These findings reshape our understanding of life in impoverished soils, underscore the hidden resilience of microbial communities, and offer clues for restoring degraded ecosystems in the face of global change.

## INTRODUCTION

Ecosystem retrogression is a phenomenon observed in various ecosystems globally^1^, ^2^, ^3^. This process occurs when an ecosystem experiences prolonged periods (thousands to millions of years) without significant rejuvenating disturbances. Retrogression is associated with reduced nutrient availability due to prolonged weathering and soil development, leading to nutrient losses over time^1^, ^2^. Among these, phosphorus (P) becomes particularly limiting to ecosystem primary productivity, as it is either leached from the system or transformed into occluded and recalcitrant forms that are not readily available to plants^3^, ^4^, ^5^. Nitrogen (N) availability can also decline during retrogression, as P limitation constrains N_2_ fixation and N becomes bound in plant litter and humus as polyphenolic complexes^6^, ^7^. As a result of retrogression, plant biomass declines, net primary productivity decreases, plant litter decomposition rates decrease, and soil aggregates become less stable, leading to reduced porosity and water retention^2^, ^8^.

In retrogressed environments, prolonged nutrient depletion drives shifts in plant strategies, favoring species that conserve resources and invest in structural and chemical defenses, such as overproducing phenolic compounds or accumulating silica in tissue^9^, ^10^, ^11^. These adaptations, along with changes in plant community composition and litter quality, reshape the input of organic matter into soil and alter nutrient cycling dynamics^12^. As a result, the structure and function of the soil food web are profoundly modified^13^. These cascading effects ultimately impact soil microbial communities, leading to long‐term declines in their diversity, abundance, and functional potential^8^, ^14^, ^15^.

Studies across global chronosequences have provided important insights into microbial responses to retrogression. At the Jurien Bay chronosequence in southwestern Australia, which spans over two million years of soil development, bacterial and fungal abundances declined in parallel with reductions in P availability and mycorrhizal activity^14^, ^15^. Similar shifts have been observed in the Haast dune system in New Zealand, where a transition from bacterial to fungal dominance was observed across a ∼3900‐year chronosequence, with increased bacterial–fungal network connections^16^. A pyrosequencing‐based study in the same region further revealed that bacterial community structure changed significantly with dune age and was strongly associated with declines in P, pH, and carbon (C):N ratios^17^. Extending to a 120,000‐year time scale, Jangid et al. examined a chronosequence near Franz Josef Glacier in western New Zealand, observing rapid changes in microbial community composition early in succession, followed by stabilization during retrogression, shaped by shifting plant–microbe feedback^18^.

Another well‐studied long‐term soil chronosequence is the Ecological Staircase of Mendocino, California, USA. This system features marine terraces formed from the same parent material, with soils ranging from young, nutrient‐rich near the coast to old, highly acidic, weathered podzols further inland^19^. This creates a natural soil fertility and acidity gradient in which the availability of P and N declines with age^20^, ^21^. The young terraces support a productive mixed conifer forest, while the older sites host a Pygmy Forest^22^. Trees in the Pygmy Forest are rich in polyphenols, which increase nutrient retention, detoxify soil aluminum, and affect N availability^6^, ^23^.

Microbiological studies on these terraces have shown significant differences in microbial community composition and a decline in bacterial and fungal diversity from younger to older terraces^24^, ^25^, ^26^. Studies using natural abundance N stable isotope patterns indicate that mycorrhizal fungi and N‐fixing bacteria provide most of the plant‐accessible N in the Ecological Staircase retrogressive‐phase terraces^27^. Additionally, it has been shown that in older terraces, ericoid mycorrhizal plants obtain significantly more N from mycorrhizal transfer than ectomycorrhizal plants, explaining the greater ericoid mycorrhizal colonization at older terraces^28^. Finally, studies of carbon use efficiency (CUE), the proportion of substrate C converted into microbial biomass, demonstrated higher CUE in the older terraces, which was correlated with soil organic matter content, litter stoichiometry, and pH, suggesting that microbial communities at older sites are energy‐limited and likely immobilizing nutrients^29^.

Microbial diversity is positively correlated with ecosystem functions^30^, so changes in microbial community composition due to ecosystem retrogression are likely to have significant implications for numerous ecosystem services. Building on the extensive foundational research carried out at the Ecological Staircase, we examined how microbial communities across domains (DNA viruses, prokaryotes, fungi, and protists) respond to ecosystem retrogression. We hypothesized that ecosystem retrogression enhances microbial connectivity and cross‐domain associations, thereby sustaining ecosystem stability despite nutrient depletion, with fungi playing a central stabilizing role due to their adaptive capacity under nutrient‐limiting conditions. Gene‐centric analyses revealed a marked enrichment of fungal genes associated with P mobilization at older sites. Metagenome‐assembled genomes (MAGs) further characterized many of these fungi as ericoid mycorrhizal fungi with high genetic potential for nutrient acquisition under extreme nutrient limitation. The pivotal role of fungi in sustaining microbial network structure was evident in network analyses, where their removal triggered a pronounced collapse in network robustness, underscoring their critical contribution to ecosystem resilience. Genomic predictions of phage–host associations suggest potential links between bacteriophages and bacterial taxa linked to P cycling, adding an overlooked viral dimension to nutrient regulation in retrogressed soils. Despite reductions in microbial diversity and abundance, cross‐domain microbial connectivity strengthened with soil age, supporting the view that retrogression leads to microbial reorganization and increased genetic potential for functional resilience.

## RESULTS

### Retrogression significantly alters microbial community composition and diversity

**Figure 1:**
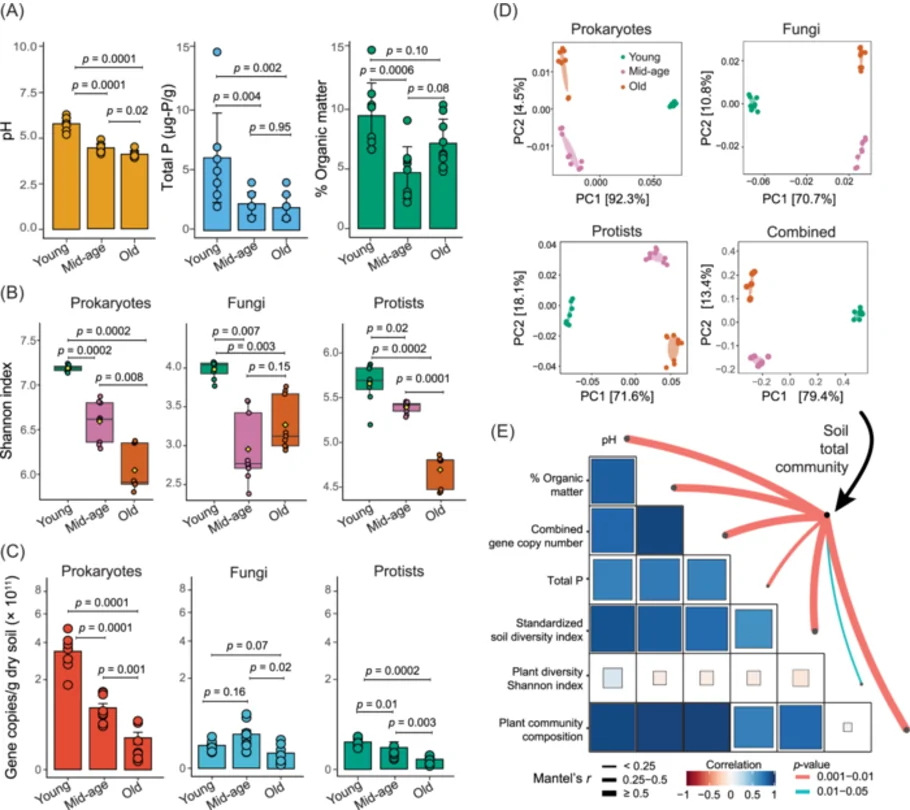
Ecosystem retrogression across marine terraces at the Ecological Staircase (Mendocino, California, USA) alters the diversity, abundance, and composition of major soil microbial groups. (A) Selected soil properties across young, mid‐age, and old terraces. (B) Alpha diversity (Shannon index) of prokaryotes, fungi, and protists. (C) Microbial abundances expressed as gene copies per gram of dry soil. The *y*‐axis is square‐root‐transformed for visualization. Significant differences were assessed using ANOVA, followed by Tukey's HSD test. (D) Weighted UniFrac ordination showing community composition by terrace and Bray–Curtis ordination of the combined dataset representing the soil total community. (E) Relationships between total community composition (Bray–Curtis) and environmental variables. Edge width represents Mantel test *r*‐values; edge color indicates significance. Pairwise Pearson correlations are shown using a color gradient, with square size indicating correlation strength. Sample size per terrace, *n* = 9. Significant differences were assessed using analysis of variance (ANOVA).

To assess the impact of soil retrogression on belowground microbial biodiversity, community structure, and genetic potential, we analyzed cross‐domain microbial communities along a marine‐derived soil chronosequence. This chronosequence serves as a natural space‐for‐time substitution, in which soils of different ages represent successive stages of long‐term ecosystem development, allowing inference of ecological and biogeochemical trajectories operating over tens of thousands of years. Soil properties varied significantly among terraces (Figures 1A and S1). The young terrace showed the highest pH, total P, soil organic matter (SOM), total organic carbon (TOC), and total N. In contrast, the mid‐age and old terraces were more acidic and nutrient‐depleted. Notably, TOC decreased at the mid‐age terrace, but increased again in the oldest terrace (Table S1). Microbial alpha diversity, measured using the Shannon index, declined significantly across terraces for all groups (Tukey's test, *p* < 0.05), indicating a loss of diversity with increasing retrogression (Figure 1B). Similarly, prokaryotic abundance declined by approximately 74.8% from the young to mid‐age terrace (*p* < 0.001) and by approximately 70.9% from mid‐age to old terrace (*p* < 0.01). Protist abundance followed a similar pattern, decreasing by approximately 67.1% from young to mid‐age (*p* < 0.001) and by 64.6% from mid‐age to old (*p* < 0.01). In contrast, fungal abundance peaked at the mid‐age terrace, increasing by 117% relative to the young terrace, but did not differ significantly between the mid‐age and old terraces (Figure 1C).

Community structure was strongly shaped by terrace age (Figure 1D). A permutational analysis of variance revealed significant terrace age effects, explaining 95.2%, 82.0%, and 80.8% of the variation in prokaryotic (*df* = 2, *F* = 232, *p* = 9.9 × 10^−5^), protist communities (*df* = 2, *F* = 82, *p* = 9.9 × 10^−5^), and fungal communities (*df* = 2, *F* = 48, *p* = 9.9 × 10^−5^), respectively. A multivariate analysis of the combined dataset (Bray–Curtis dissimilarity) also showed that terrace age accounted for 86% of the total variation in community composition (*df* = 2, *F* = 73.7, *p* = 9.9 × 10^−5^) (Figure 1D). Inclusion of soil and vegetation covariates (SOM, total N, TOC, total P, plant β‐diversity, and plant Shannon index) increased model fit to 91.4% (*df* = 8, *F* = 22.5, *p* < 0.001). Terrace age remained a significant independent predictor after accounting for environmental co‐gradients (*R* ^2^ = 0.109, *F* = 10.75, *p *= 0.0001). Furthermore, variance partitioning revealed that terrace age uniquely explained 13% of the total variance, environmental covariates explained 2%, and their shared effects explained 72% (Figure S2). These results confirm that microbial differentiation across terraces reflects broad, terrace‐level processes associated with long‐term ecosystem retrogression.

To further identify the environmental variables most closely associated with community turnover across terraces, we performed Mantel tests between Bray–Curtis community dissimilarities and soil or vegetation distance matrices. Microbial community composition was strongly correlated with key soil and plant properties known to shift during retrogression. The strongest correlations were observed for soil pH (*r* = 0.86, *p* = 0.001), plant community composition (*r* = 0.81, *p* = 0.001), total N (*r* = 0.67, *p* = 0.001), and total P (*r* = 0.45, *p* = 0.001) (Figures 1E, and S3). Additional significant correlations included total microbial abundance (*r* = 0.73), standardized soil diversity index (*r* = 0.78, *p* = 0.001), SOM (*r* = 0.26, *p* = 0.001), and plant diversity (*r* = 0.15; *p* = 0.03). These relationships highlight the coupling between microbial community composition and the chemical and biological gradients that co‐vary with terrace age.

### Microbial communities adapt to retrogression by enhancing cross‐domain network resilience

**Figure 2:**
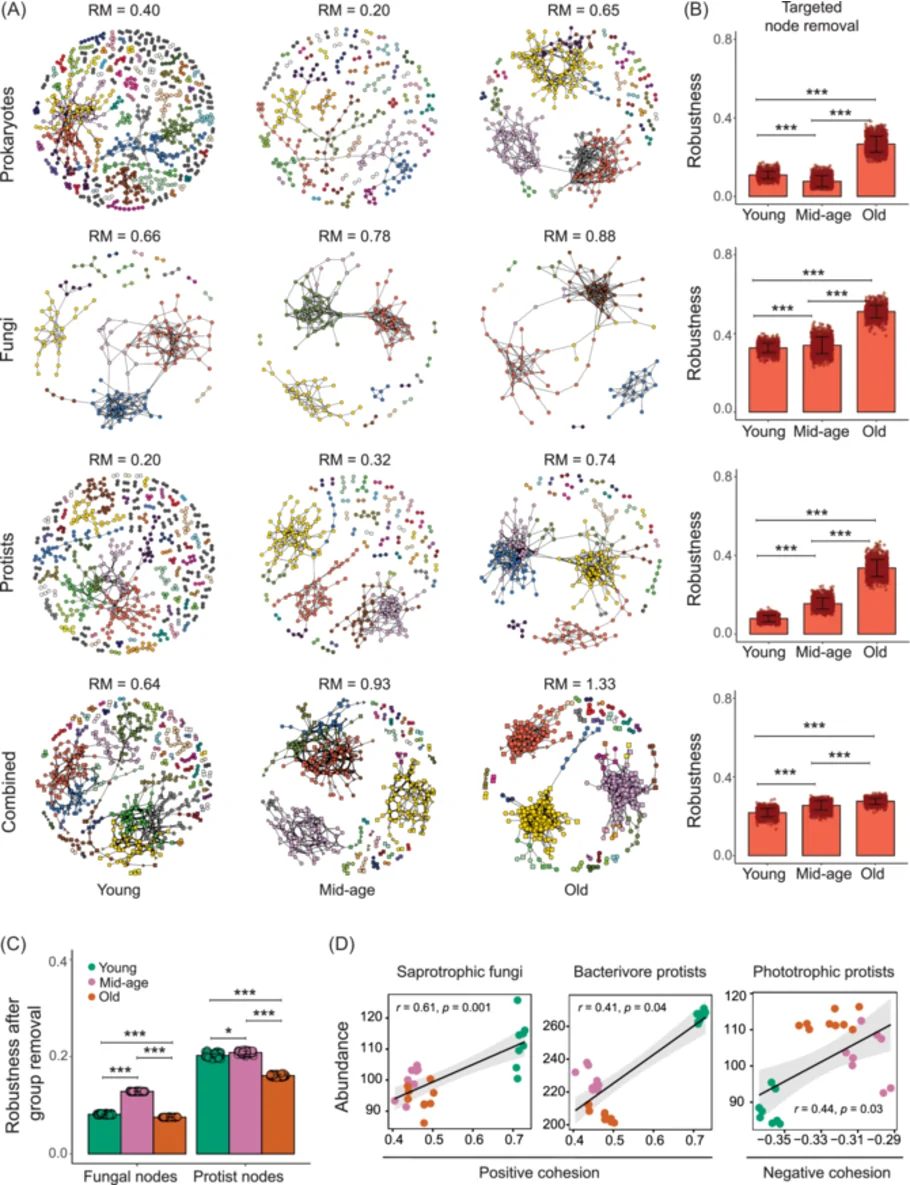
Ecosystem retrogression alters network complexity and community cohesion. (A) Network structures differed across terraces for individual domains and combined microbial groups. Networks were reconstructed using random matrix theory (RMT)‐based co‐occurrence models from biological replicates (*n* ≥ 8 per terrace). Nodes represent operational taxonomic units (OTUs) or zero‐radius OTUs (ZOTUs), and edges represent significant correlations. Modules (subpopulations) are shown in random colors. RM, relative modularity. (B) Network robustness, measured as the normalized area under the curve (AUC) under targeted node removal across microbial groups and terraces. High‐degree nodes were removed sequentially, with ties resolved randomly. (C) Network robustness after targeted removal of protists and fungal nodes across terraces. Node removal was randomized across iterations to account for variation. Significant differences were calculated using the Kruskal–Wallis test, followed by Dunn's test with Benjamini–Hochberg *p*‐value correction. (D) Pearson correlations between calculated cohesions and the abundance of predicted functional groups with *p*‐values corrected using Benjamini–Hochberg correction. Sample size per terrace, *n* = 9. *p < 0.05 and ***p < 0.001.

We reconstructed molecular ecological networks to evaluate the effects of retrogression on the structure, resilience, and complexity of soil microbial communities. Networks were built for individual and combined microbial groups (Figure 2), and their topological properties were characterized (Table S2). Modularity, which quantifies the degree to which a network is compartmentalized into subcommunities, was among the network properties evaluated. Because network size and connectivity varied among terraces, we calculated Relative Modularity (RM), a normalized measure that enables meaningful comparisons across networks. RM varied with retrogression across all microbial groups, reaching its highest values in the oldest terraces. The magnitude and trajectory of this increase differed by group: RM increased steadily for fungi (0.66 → 0.88) and protists (0.20 → 0.74), while prokaryotic RM followed a U‐shaped trend, decreasing at mid‐age but rebounding sharply in the oldest terrace (0.40 → 0.65). The combined network showed a consistent increase in modular organization (0.64 → 1.33), indicating stronger compartmentalization at the whole‐community level. Increased RM has been associated with higher community stability^31^, ^32^, suggesting that microbial communities in retrogressed soils maintain structural stability through enhanced modular organization.

Network stability was further assessed by measuring robustness to random and targeted node removal (Figures S4 and 2B). Under random node removal, network robustness was significantly higher in older terraces across all groups (Dunn's test, *p *< 0.001), further suggesting greater structural stability with retrogression (Figure S4). The combined network showed the highest robustness at the mid‐age terrace, followed by older and young terraces (Dunn's test, *p* < 0.001). This may indicate that older microbial communities are better adapted to disturbances. Targeted node removal analysis confirmed higher robustness in older terraces, preserving network integrity despite high‐degree node loss (Dunn's test, *p* < 0.001) (Figure 2B). Removal of fungal nodes had a significantly greater impact on robustness than protist removal (Dunn's test, *p* < 0.001; Figure 2C), highlighting fungi's key role in ecosystem stability. Network vulnerability, measured as maximum efficiency loss from single‐node deletion, was the lowest in older terraces, further supporting their enhanced resilience (Table S3).

We calculated the cohesion index to assess community complexity. Positive cohesion (putative cooperative interactions) declined with soil age across all microbial groups and combined networks, reaching the lowest levels in the mid‐age terrace (Figure S5). Negative cohesion (putative competitive interactions) was lower in fungal and combined networks, indicating greater cooperation, but remained stable in prokaryotic networks. In protists, negative cohesion increased with soil age (Dunn's test, *p* < 0.001), while younger terraces showed higher negative cohesion in combined networks (Dunn's test, *p *< 0.001). Associations between micro‐eukaryotic functional groups and cohesion (Figure 2D) revealed that saprotrophic fungi (*r* = 0.61, *p* < 0.01) and predatory/omnivorous protists (*r* = 0.41 and 0.44, *p* < 0.05) significantly contributed to positive cohesion, enhancing microbial interactions. However, these groups were also linked to higher negative cohesion, likely due to resource competition. Phototrophic protists were an exception, showing increased abundance in older terraces with open canopies, where their ability to fix C may alleviate competitive pressures, reflected in a significant reduction in negative cohesion (*r* = 0.44, *p* < 0.05).

### Different ecological processes influence community assembly as ecosystems retrogress

**Figure 3:**
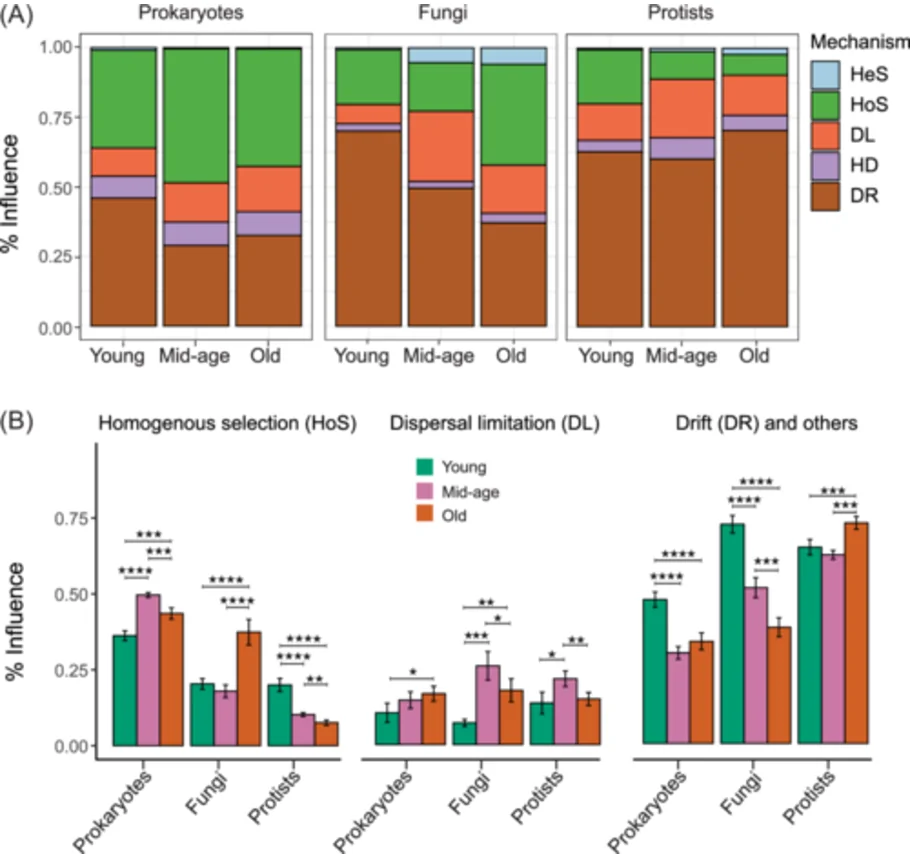
Relative influence of different ecological processes in shaping soil community assembly during ecosystem retrogression. (A) Homogeneous selection (HoS), drift (DR), and dispersal limitation (DL) were dominant processes across microbial groups and terraces. HD, homogenizing dispersal; HeS, heterogeneous selection. (B) Differences in the contribution of ecological processes across terraces for each microbial group. Significance was assessed using bootstrapping with 1000 replications. *p < 0.1, **p < 0.05, ***p < 0.01, and ****p < 0.001.

We examined ecological processes shaping community assembly across terraces to identify mechanisms driving changes in microbial community and network structure. Homogeneous selection (HoS), dispersal limitation (DL), and drift (DR) were the dominant factors influencing cross‐domain community assembly (Figure 3A).

HoS significantly shaped prokaryotic assembly in the mid‐age terrace and fungal communities in the old terrace (*p* < 0.001), favoring more similar communities under abiotic and biotic constraints^33^. Protist communities were also shaped by HoS (*p* < 0.001), particularly in the young terrace, where higher prey abundance and diversity favor biotic interactions (Figure 1B,C).

DL, reflecting restricted microbial movement and establishment, was significant for prokaryotes and fungi in older terraces, and for protists in the mid‐age terrace (Figure 3A,B). Lower soil water‐retention capacity, a characteristic of retrogression, in the older terraces may explain its stronger dispersal constraints (Table S1).

DR, a stochastic process involving weak selection, diversification, and low dispersal, dominated prokaryotic and fungal assembly in the young terrace, but had a greater influence on protist communities in retrogressed sites (Figure 3A,B).

### Fungal functional genetic potential is amplified in retrogressed ecosystems

Metagenome sequencing and assembly were conducted to define changes in microbial functional potential. Using a gene‐centric approach, we identified, annotated, and determined the abundance of prokaryotic genes associated with P and N mineralization, N fixation, and plant biomass degradation across the terraces. We also reconstructed MAGs to identify prokaryotic populations potentially performing these ecosystem services.

**Figure 4:**
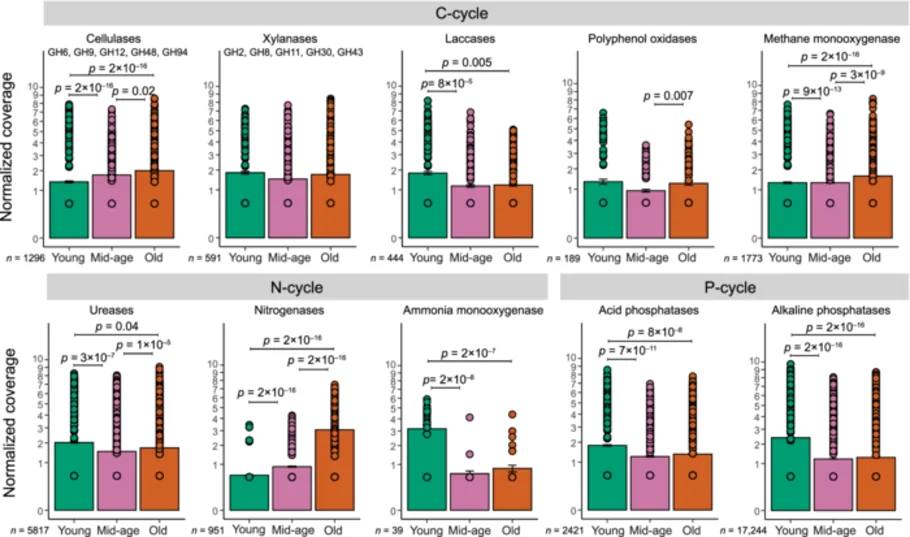
Normalized abundances of putative prokaryotic genes involved in C, N, and P cycling across terraces. Abundances were variance‐stabilized using DESeq. 2 and square‐root‐transformed for visualization. Significant differences were calculated using the Kruskal–Wallis test, followed by Dunn's test with Benjamini–Hochberg *p*‐value correction.

Analysis of gene distribution indicated that nitrogenase genes were significantly more abundant in the older terraces (Dunn's test, *p* < 0.001) (Figure 4). Twelve MAGs (Figure S6), including *Beijerinckia*.4764, *Rhodomicrobium*.2215, and *Rhodoblastus*.2093, carried nitrogenase genes and were highly abundant in the older terrace. In contrast, urease, ammonia monooxygenase, laccases, and phosphatase genes were significantly more abundant in the younger terrace (Dunn's test, *p* < 0.05) (Figure 4). Eighty‐seven MAGs spanning multiple bacterial phyla, including *Acidobacteriota*, *Actinomycetota*, and *Chloroflexota*, contained urease genes (Figure S6). One archaeal MAG, identified as *Nitrososphaeraceae*.4919, carried genes coding for ureases and ammonia monooxygenases.

Given the limited P availability in retrogressed ecosystems, we hypothesized that phosphatase genes would be more abundant in older terraces, where these elements have become less available due to interactions with minerals and organic matter. However, our findings contradicted this hypothesis: prokaryotic alkaline and acid phosphatase genes were significantly more abundant in the young terrace (Dunn's test, *p* < 0.001). Out of 321 MAGs, 189 contained genes for alkaline phosphatases and 48 contained genes for acid phosphatases, with 29 MAGs containing both, including *Phenylobacterium*.162_sub, *Longimicrobium*.841, *Sphyngopyxis*.2234, *Reyranella*.1239_sub, and *Minicystis*.1473 (Figure S6).

**Figure 5:**
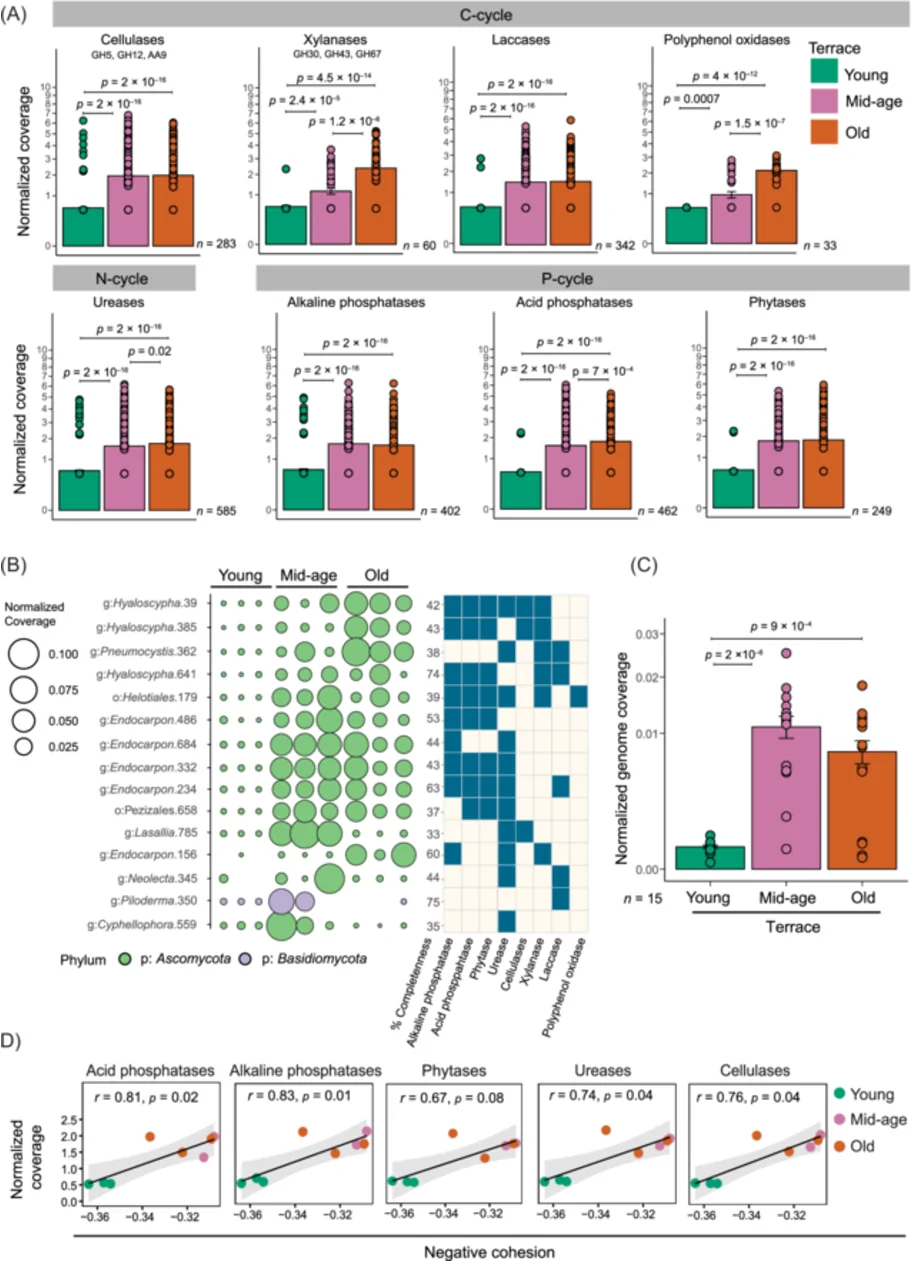
Effects of ecosystem retrogression on fungal communities and their nutrient cycling genes. (A) Abundance of putative fungal genes linked to C, N, and P cycling across terraces. (B) Distribution of fungal MAGs and presence/absence patterns of selected nutrient cycling. (C) Total fungal MAG coverage by terrace. Significant differences were calculated using the Kruskal–Wallis test, followed by Dunn's test with Benjamini–Hochberg *p*‐value correction. (D) Significant Pearson correlations between fungal gene abundance and negative cohesion. MAGs, metagenome‐assembled genomes.

We also analyzed the abundance patterns of fungal genes, as fungi are hypothesized to play a critical role in the functioning of retrogressed ecosystems and may contribute to decreased competitive associations, as observed in our robustness analysis on the networked communities (Figure 2D). Our findings showed a significant increase in the abundance of fungal genes coding for cellulases and xylanases in the older terraces (Dunn's test, *p* < 0.001) (Figure 5A). Prokaryotic genes coding for cellulases were also more abundant in older terraces, while prokaryotic genes coding for xylanases were evenly distributed across all terraces (Figure 4). Notably, our results demonstrated that fungal genes for phytases, acid and alkaline phosphatases, and ureases were significantly more abundant in the mid‐age and older terraces than in the younger site (Dunn's test, *p* < 0.001) (Figure 5A). This provides clues to the crucial role of fungi in mobilizing P and N in retrogressed ecosystems, potentially contributing to the higher network connectivity observed in the combined molecular networks.

Retrogressed ecosystems, such as the older sites at the Ecological Staircase, harbor resource‐conserving plant communities that overproduce polyphenols to inhibit herbivory and resist degradation. Our results showed that fungal genes coding for laccases and polyphenol oxidases were significantly more abundant in retrogressed soils (Dunn's test, *p* < 0.001) (Figure 5A), while those of prokaryotic origin are more abundant in the younger terrace (Figure 4). This suggests that fungi play a key role in P and N availability, and impact the fate of plant material in retrogressed ecosystems, especially aromatic compounds derived from lignin.

Correlations between gene abundances and soil properties revealed strong patterns consistent with nutrient depletion along the retrogression gradient (Figure S7 and Table S4). Fungal phosphatase‐, phytase‐, and urease‐encoding genes were negatively correlated with total P, total N, and pH, indicating higher abundance in acidic, nutrient‐poor soils (*r* = –0.7 to –0.9, *p* < 0.05). In contrast, the same genes of prokaryotic origin showed positive correlations with the same soil parameters (*r* = 0.7–0.96, *p* < 0.05). Nitrogenase gene abundance increased with decreasing pH, total N, and total P, although only significantly with pH (*r* = –0.76, *p* = 0.03). Fungal and prokaryotic genes for C degradation (cellulases, xylanases, laccases, and polyphenol oxidases) were similarly inversely related to nutrient levels and pH, underscoring the tight coupling between C decomposition, acidification, and nutrient depletion. After accounting for covarying soil factors, most correlations lost significance (Figure S7 and Table S4), suggesting that shared environmental gradients drive many apparent relationships between gene abundance and nutrient pools.

To disentangle these interrelated effects and infer causal pathways, we used a piecewise structural equation model (piecewise SEM) to quantify both direct and indirect drivers of microbial functional potential. In the piecewise SEM, we focused on pH, total N, and total P to maintain parsimony, given our total number of observations. These soil variables showed the strongest associations with gene abundances in the bivariate analysis, which represent the dominant biogeochemical axis of retrogression (Figure S8 and Text S1). The model (Fisher's *C* = 56.4, *df* = 42, *p* = 0.067) explained most variance in community composition (*R* ^2^ = 0.95) and functional gene indices (*R* ^2^ = 0.82–0.99). Total N was negatively associated with microbial community structure (*β* = –1.35, *p* = 0.05), indicating that declining N availability drives compositional shifts. In contrast, total P had strong positive effects on fungal phosphatase (acid and alkaline) and urease indices, as well as bacterial urease potential (*β* = +0.41 to 0.51, *p* < 0.05). Soil pH exerted direct negative effects on both fungal and bacterial phosphatase indices, and on the bacterial urease index (*β* = –0.56 to –3.67, *p* < 0.05), the latter also increasing with total N (*β* = +6.04, *p* < 0.05). Community composition mediated contrasting domain‐level responses, enhancing fungal while suppressing bacterial nutrient cycling potential (*β* = –3.5 to +5.2, *p* < 0.05). Overall, the results suggest that long‐term N depletion indirectly reorganizes microbial communities, while P and pH act as direct environmental constraints on microbial functional potential, together promoting a shift from bacterial to fungal dominance in nutrient acquisition strategies during ecosystem retrogression.

Microeukaryotic genome‐resolved metagenomics identified key fungal players potentially involved in P mobilization, ammonia production from urea, and lignocellulose degradation. Fifteen fungal MAGs were reconstructed, with completions ranging from 33% to 75% (contamination <10%) (Figure 5B). These MAGs were more abundant in the mid‐age (Figure 5C), consistent with quantitative PCR (qPCR) results showing higher fungal abundance on the same terrace (Figure 1C). Among the MAGs, *Hyaloscypha*.39 contained genes for alkaline and acid phosphatases, ureases, cellulases, and xylanases. Most other fungal MAGs also had genes for both phosphatases, except for *Endocarpon*.684 and *Endocarpon*.156 (alkaline only) and *Pezizales*.658 (acid only). Urease genes were prevalent in most fungal MAGs, except for *Hyaloscypha*.385 and 641, *Endocarpon*.486, and *Piloderma*.350. Laccase genes were found in *Pneumocystis*.362, *Hyaloscypha*.641, *Endocarpon*.234, *Neolecta*.345, and *Piloderma*.350 (Figure 5B).

An analysis was conducted to explore the relationship between the normalized abundance of prokaryotic and fungal functional genes and community cohesion (Figures S9 and 5D). Positive cohesion was strongly correlated with the prevalence of several prokaryotic enzymes, including alkaline and acid phosphatases (*r* = 0.85 and 0.88, respectively, *p* < 0.01), ammonia monooxygenases, prokaryotic laccases, polyphenol oxidases (*r* = 0.75 and 0.77, *p* < 0.05), and prokaryotic ureases (*r* = 0.92, *p* < 0.01). In contrast, negative cohesion was inversely correlated with the prevalence of most putative fungal functional genes, with higher gene abundances corresponding to less negative cohesion (Figure 5D). These findings further underscore the pivotal role of fungi in maintaining connectivity within microbial communities in retrogressed soils.

### Viral diversity declines with soil retrogression and may influence P availability

In total, 1636 DNA viruses were identified from the metagenomes, including 1621 *Caudoviricetes* (phages) and 15 *Nucleocytoviricota* (giant viruses). Among the *Nucleocytoviricota*, seven were *Algavirales* (viruses of micro‐algae), five were *Imitervirales* (viruses of protists), and three were *Pimascovirales* (viruses of arthropods).

**Figure 6:**
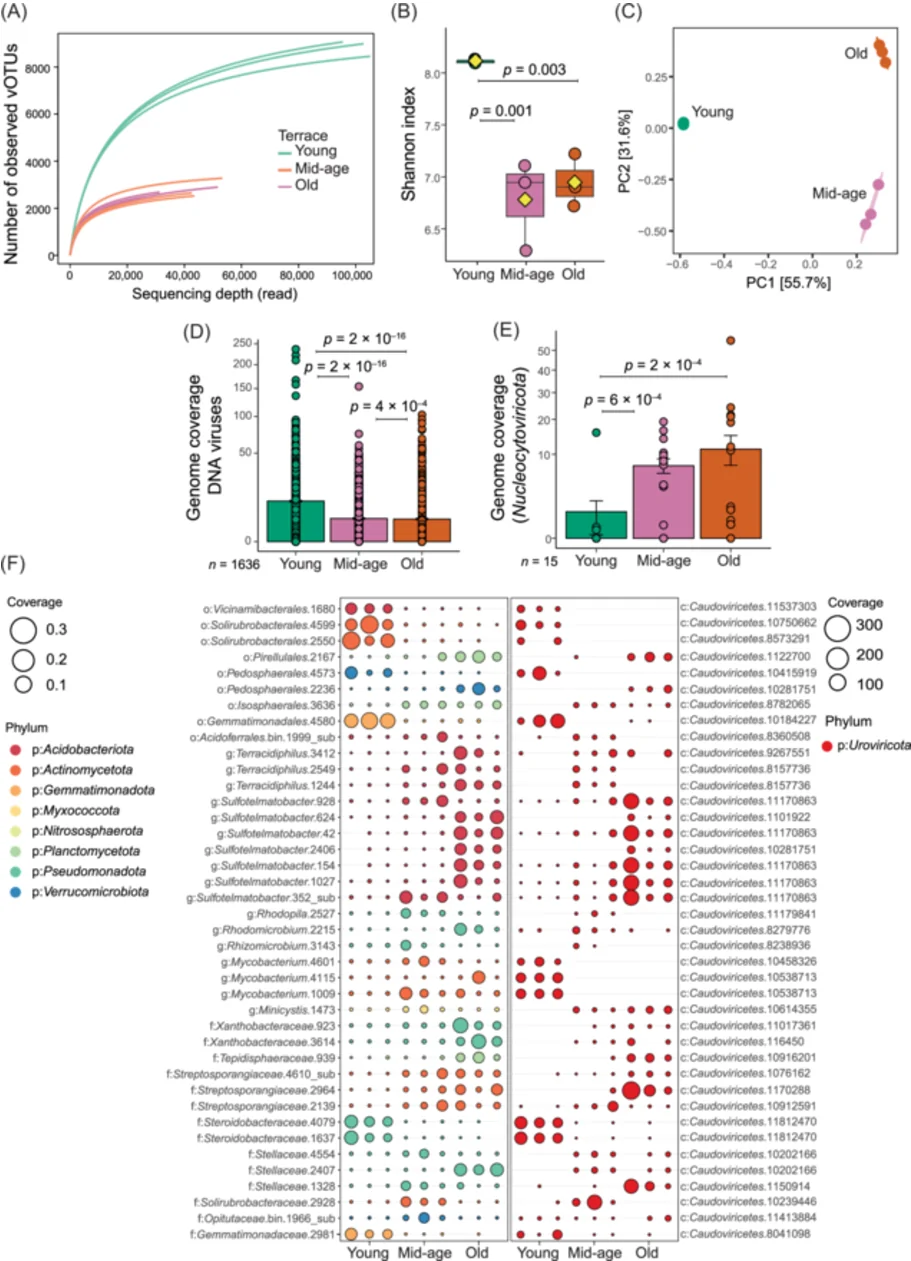
DNA viruses are also affected by ecosystem retrogression, likely due to host diversity changes. (A) Accumulation curves for DNA viruses among terraces based on mapped metagenome reads to identified viral OTUs (vOTUs). (B) Shannon diversity index calculated on dereplicated viral MAGs. Significant differences were assessed using ANOVA, followed by Tukey's HSD test. (C) Ordination plots showing the differences in viral community composition. (D, E) Total viral MAG abundance (D) and *Nucleocytoviricota* MAG abundance (E) across terraces. Significant differences were calculated using the Kruskal–Wallis test, followed by Dunn's test with Benjamini–Hochberg *p*‐value correction. (F) Normalized genome coverage patterns of prokaryotic MAGs and their corresponding phage parasite.

Accumulation analysis confirmed that metagenome‐mapped reads corresponding to identified DNA viruses effectively sampled the analyzed soil viral communities (Figure 6A). The Shannon diversity of DNA viruses significantly declined from the young to the older terraces (Tukey's test, *p* < 0.001), with comparable diversity levels observed between the mid‐age and old terraces (Figure 6B). Community composition analysis showed a clear differentiation of DNA viral communities per terrace, showing the marked effect of retrogression across life domains (Figure 6C). In a similar trend to what was observed with prokaryotes, fungi, and protists, the normalized abundance of DNA viruses decreased progressively from the young to the older terraces (Dunn's test, *p* < 0.001) (Figure 6D). *Caudoviricetes* were significantly more abundant in the young terrace (Dunn's test, *p* < 0.001), coinciding with its higher prokaryotic diversity and abundance. In contrast, *Nucleocytoviricota*, including members of *Algavirales* and *Pimascovirales*, were more prevalent in the older terraces (Dunn's test, *p* < 0.001), aligning with the increased abundance of phototrophic protists in these terraces (Figure 6E).

Potential hosts were identified for 198 *Caudoviricetes* using the Integrated Phage‐Host Prediction (iPhoP) database: 39% could infect *Actinomycetia*, 27% could infect *Alphaproteobacteria*, 16% could infect *Terriglobia*, 4% could infect *Gammaproteobacteria*, and 3% could infect *Thermoleophilia*. Hosts were identified for 63 *Caudoviricetes* among our reconstructed prokaryotic MAGs with confidence scores >90% and host and phage prevalence mirroring each other (Figure 6F).

We also analyzed auxiliary metabolic genes (AMGs) involved in P metabolism among phages. The phage *Caudoviricetes*.10952585 carried the *phoD* gene, which encodes an alkaline phosphatas^34^, as confirmed by protein structure modeling (Phyre2; 100% confidence; 98% coverage; and 18% amino acid identity). Phosphatase activity of *phoD* AMGs has been experimentally confirmed in other systems^35^, suggesting a potential, though unconfirmed, contribution to host P turnover under low‐P conditions. While no direct host association was identified among the reconstructed MAGs or iPHoP predictions, phylogenetic evidence suggests horizontal acquisition of *phoD* from a *Pseudomonadota* lineage (Figure S10).

**Figure 7:**
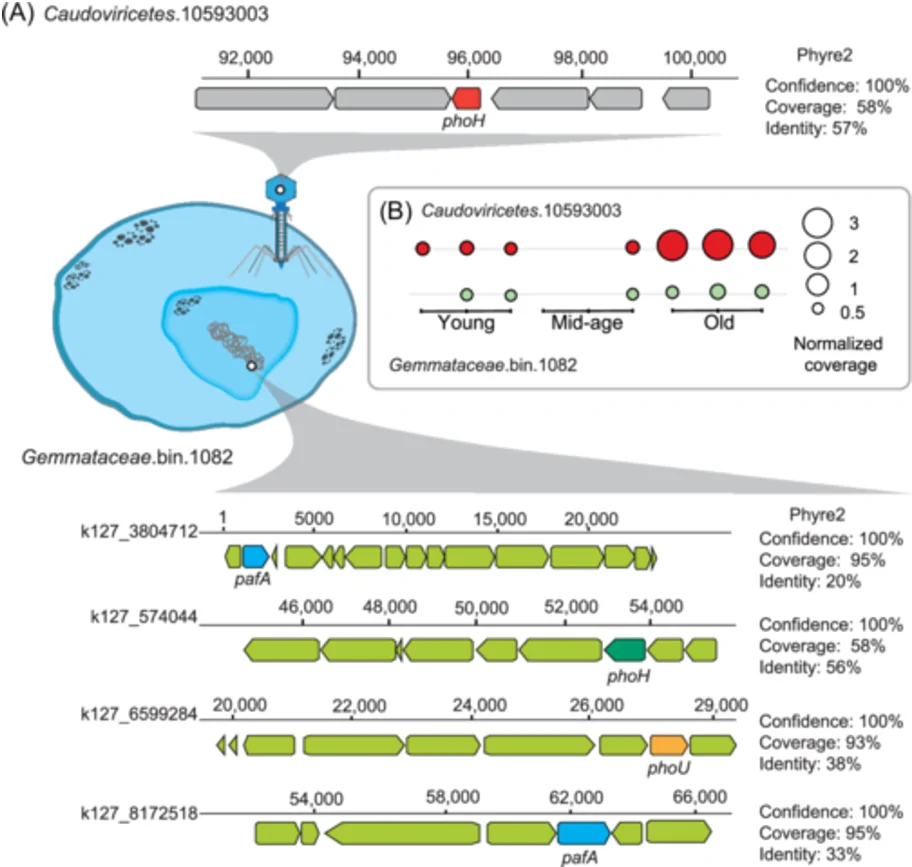
Genomic context and abundance of P‐mobilization genes in a *Caudoviricetes* phage and its putative *Gemmataceae* host across a soil chronosequence. (A) Partial genome maps for phage and the corresponding *Gemmataceae* host showing genes involved in P mobilization. Predicted proteins were further corroborated in Phyre2. (B) Abundance patterns for phage and host across terraces. Notice the coordinated prevalence patterns between phage and host. *phoH* and *phoU*, phosphorus uptake‐regulating genes; and *pafA*, phosphate‐insensitive phosphatase.

Another phage enriched in the older terraces, *Caudoviricetes*.10593003, encoded a phosphate starvation‐inducible protein PhoH (Phyre2; 100% confidence; 58% coverage; 57% amino acid identity), a Pho regulon‐associated protein linked to bacterial responses to low‐phosphate conditions^36^ (Figure 7A). Host prediction by iPHoP linked this phage to *Gemmataceae*.1082 with a 93.9% confidence score. The Gemmataceae MAG encodes two phosphatase‐insensitive phosphatases (PafA), along with PhoH and PhoU, consistent with an adaptive phosphorus‐acquisition and homeostasis system^37^. Coverage‐based co‐occurrence analysis supported the predicted association between *Caudoviricetes*.10593003 and *Gemmataceae*.bin.1082. Within‐sample correlations across all replicates were significant (*r* = 0.73, *p* = 0.026), and terrace‐level mean coverages showed a perfect linear relationship (*r* = 1.00, *p* = 0.007). These results indicate consistent co‐enrichment of the phage and its host across retrogression stages (Figure 7B). Together, these results support a possible phage–host association influencing P metabolism, although the precise functional and mechanistic interactions remain to be experimentally validated.

## DISCUSSION

Over extended time scales, and in the absence of disturbances that reset succession, ecosystems undergo retrogression, a stage marked by reduced primary productivity, slower decomposition, and diminished nutrient cycling^3^, ^38^, ^39^. Retrogressive ecosystems are further characterized by declining plant biomass, low productivity, and the dominance of resource‐conservative plant genotypes that produce slowly decomposing foliage rich in phenolic compounds^7^, ^9^, ^40^. Because such transitions unfold over tens of thousands of years, they cannot be directly observed within the lifespan of investigators. To address this, we applied a space‐for‐time substitution using a well‐characterized marine terrace chronosequence, a widely established approach in ecosystem ecology for inferring long‐term trajectories. Within this framework, we integrated amplicon sequencing, gene‐centric and genome‐resolved metagenomics, and co‐occurrence network modeling to test how microbial communities maintain resilience and functional capacity during advanced ecosystem retrogression. This multi‐omics approach not only extends earlier findings from the Ecological Staircase but also provides new mechanistic insights into the assembly, adaptation, and functional strategies of cross‐domain microbial communities under severe nutrient limitation.

### Ecosystem retrogression impacts all soil microbial domains

Our study demonstrates a marked decline in microbial diversity and abundance from young to old terraces, highlighting how soil retrogression imposes intense selective pressures that restructure belowground communities (Figure 1). This trend was consistent for both prokaryotes and protists, which showed significant reductions in diversity and abundance with decreasing nutrient availability (Figure 1A–C). Similarly, the diversity and abundance of bacteriophages identified through metagenomics decreased in the mid‐age and old terraces (Figure 6B,D,E), likely reflecting diminished host diversity and reduced P availability, given the high P demands of viral replication^41^, ^42^. Fungal communities did not follow a linear decline (Figures 1B,C and 5B,C). The higher abundance of fungi observed in the retrogressed sites may be related to the significant investment by plants in mycorrhizae and the need for fungi to adopt exploration strategies in nutrient‐poor soils^24^, ^43^. Similar microbial shifts have been reported across globally distributed chronosequences despite differing methodological frameworks. For instance, amplicon‐based studies in New Zealand's Haast and Franz Josef dunes^16^, ^17^, ^18^, and lipid biomarker analysis in the subtropical dunes of Georgia^44^, report bacterial declines and fungal increases consistent with retrogressive nutrient limitation. Despite differing site characteristics and analytical approaches, these studies converge on similar domain‐specific patterns, reinforcing that a combination of physicochemical and biological factors drives microbial trajectories across retrogressive systems.

In our study, all microbial groups, including DNA viruses, showed distinct community compositions across terraces, underscoring strong niche differentiation driven by environmental changes associated with ecosystem retrogression (Figures 1D and 6C). These patterns align with previous observations in the Mendocino terraces^25^, ^26^ and reinforce findings from other chronosequences^14^, ^15^. Multivariate analysis indicated that terrace age was the main predictor of community differences across all microbial groups, explaining the largest proportion of variation even after accounting for soil and vegetation covariates. Variance partitioning further revealed that the combined influence of terrace age and environmental properties was the strongest. This reinforces the principle that long‐term pedogenic processes and contemporary abiotic and biotic gradients jointly structure microbial assemblages along the retrogression sequence^45^. Strong correlations between microbial composition, soil chemistry, and vegetation further support this interpretation, implying that terrace age encapsulates both direct and indirect pathways of ecological organization across domains of life (Figure 1E).

Analysis of community assembly mechanisms showed that homogenous selection, influenced by both biotic and abiotic factors, dominated prokaryotic and fungal communities in older terraces (Figure 3). In contrast, protist communities were primarily structured by DL and DR, likely due to restricted water availability, as previously observed in marginal soils^46^. While our study provides a snapshot of microbial communities during the summer, future work incorporating seasonal dynamics could further clarify the temporal stability of cross‐domain microbial networks under retrogression.

### Cross‐domain microbial networks become more connected with retrogression

Network analysis revealed progressive shifts in microbial community organization during ecosystem retrogression (Figure 2A). As soils aged, all networks showed greater RM, hinting at higher community stability under nutrient limitation^31^, ^32^. Robustness analyses confirmed that networks in older terraces retained higher connectivity despite reduced diversity. These results suggest that stability in nutrient‐depleted soils is maintained through intensified cross‐domain interactions (Figures 2B and 4). Fungi emerged as key structural components of these networks. Removal of fungal nodes significantly reduced network robustness across terraces, whereas removal of protist nodes had lesser impact (Figure 2C). This underscores the critical role of fungi in maintaining microbial connectivity and stabilizing ecosystems under nutrient‐limited conditions. Analysis of cohesion revealed a decline in positive cohesion from young to older terraces, indicating a reduction in cooperative interactions as ecosystems age^47^. Negative cohesion, however, displayed group‐specific trends (Figure 2D). Negative cohesion, however, displayed group‐specific trends (Figure 2D). Prokaryotic networks remained stable, which suggests that competitive and cooperative dynamics are relatively consistent across sites (Figure S5). In contrast, protist networks in older terraces showed stronger negative cohesion, potentially reflecting intensified competition for dwindling resources as retrogression progresses. Interestingly, fungal and combined networks showed a reduced negative cohesion in older sites, suggesting a shift toward cooperative behaviors, likely mediated by fungi (Figure S5). Mycorrhizal fungi, for example, extend hyphal networks that enhance nutrient distribution, bridge disconnected patches, and promote microbial cooperation^48^, ^49^. The contrasting negative cohesion trends across microbial groups suggest that ecological succession influences cooperative and competitive interactions differently, driven by selection pressures, dispersal limitation, and stochastic processes (Figure 3).

### Cross‐domain microbial genetic functional potential sustains retrogressed ecosystems

In our study, the abundance of bacterial genes coding for phosphatases remained relatively constant across the terraces, while those from fungi increased significantly in the older terraces. As P availability decreases with soil age due to its binding to soil minerals^5^, fungi may become crucial in mobilizing this essential nutrient. Fungal MAGs identified as *Hyaloscypha*, *Endocarpon*, *Pezizales*, and *Helotiales* contained genes coding for phytases, acid, and alkaline phosphatases, and are known for their capacity to establish ericoid or endomycorrhizal associations with conifers and ericaceous species, which are highly abundant in the older terraces (Figure 5B)^50^, ^51^, ^52^.

Lignocellulose‐degrading associated genes of fungal origins, such as genes encoding cellulases and xylanases, are more abundant in the older and more acidic terraces (Figure 5A), suggesting that fungi may sustain decomposition where harsh soil conditions limit other decomposers. The ability of fungi to degrade recalcitrant plant materials could be particularly important in retrogressed soils, where reduced herbivory and increased production of phenolic compounds by plants make the litter more resistant to degradation^9^, ^10^.

In parallel, the scarcity of soil N (Figure 1A) and its predominance in complex organic forms (protein–tannin complexes)^21^, ^53^, ^54^, ^55^, ^56^, may select for taxa capable of accessing organic N pools. Nitrogen stable isotope analyses at the Ecological Staircase indicate that ericoid and ectomycorrhizal fungi, together with N‐fixing bacteria, are key contributors to N supply in these nutrient‐poor soils^27^. Consistent with this, we found elevated abundances of nitrogenase and fungal urease genes in the mid‐age and older terraces within ericoid mycorrhizal fungal MAGs (Figures 4 and 5A,B).

Phototrophic protists were also notably enriched in older terraces (Figure 2D). These protists are known for their ability to fix atmospheric CO_2_, contribute to C sequestration, ameliorate soil acidity, enrich C content, and stimulate biological activity by producing exopolysaccharides and other metabolites^57^, ^58^, ^59^. Phototrophic protists range from primary producers through photosynthesis, supporting heterotrophic organisms, to tertiary predators^60^. This biological activity may enhance the overall resilience and functional stability of the microbial community by fostering beneficial interactions among different trophic levels, as suggested by their significant presence in older sites and correlation with less negative community cohesion.

Correlations between gene abundances and soil parameters revealed opposing trends between fungal and bacterial genes involved in P and N mobilization, pointing to a shift in nutrient cycling strategies during retrogression. The enrichment of genes encoding fungal phosphatases, phytases, and ureases in acidic, nutrient‐poor terraces suggests increasing reliance on fungal activity. In contrast, the decline of homologous bacterial genes may reflect a reduction in bacterial contributions to nutrient mobilization.

However, after accounting for covarying soil factors, pairwise correlations lost significance, suggesting that microbial gene distributions are shaped by the combined influence of interrelated environmental gradients rather than by any single parameter (Figure S7). This motivated the use of a piecewise SEM to disentangle direct and indirect effects of soil chemistry, community composition, and functional gene abundances. The model supports a framework in which N depletion primarily restructures microbial community composition, while P availability and pH act as direct environmental constraints on microbial functional potential, represented here by normalized gene abundances (Figure S8). Independent P fractionation data from the same chronosequence^20^ (Figure S11) show that, with increasing soil age, labile and mineral P pools decline sharply, while occluded and organic fractions accumulate. These patterns support the view that fungal enrichment reflects adaptation to increasingly recalcitrant P sources under acidic conditions. Notably, community composition mediates opposite responses across domains, enhancing the genetic potential for nutrient cycling in fungi while reducing it in prokaryotes. This divergence indicates that as soils age and acidify, microbial communities reorganize toward fungal‐dominated nutrient mobilization, consistent with the observed enrichment of fungal phosphatase, phytase, and urease genes, and with the increased prominence of fungal energy channels reported in other retrogressed ecosystems^8^, ^61^, ^62^. While these results provide a coherent causal framework, they should be interpreted as exploratory, given the limited metagenomic replication and the use of gene abundances as proxies for functional potential.

This reorganization of nutrient acquisition strategies toward fungal dominance likely reverberates across other microbial domains, including viruses and their hosts. The unique dynamics observed in our identified DNA viruses also provide insights into the microbial regulation of nutrient cycles. Notably, the decline in phage diversity with soil age paralleled the decrease in microbial diversity. In contrast, the increase in the abundance of giant viruses (*Algavirales*) paralleled the persistence of phototrophic protists in the older terraces (Figure 6E). This suggests that viruses shape microbial community structures and functions through lysogenic cycles and horizontal gene transfer, following a “kill the winner” dynamic^63^, ^64^.

Experimental evidence from P‐ and N‐limited systems supports this view. For instance, in paddy soils, phage–bacteria interactions showed reduced *Caudoviricetes* abundance and increased lysogeny under low‐P conditions^65^. Moreover, some phages carry genes such as *phoH*, which are involved in P‐starvation responses, and *phoH* expression has been directly observed following phage infection of bacterial hosts^66^. The enrichment of these P‐regulation genes in phage genomes^67^, ^68^ suggests that viral infection can modulate host P metabolism, potentially benefiting both partners under nutrient stress. Although mechanistic confirmation remains limited, our findings are consistent with the idea that bacteriophages contribute to P cycling in retrogressed soils by enhancing the mineralization capacity of their hosts.

Together, our findings highlight the interplay between microbial cooperation and competition during ecosystem retrogression, emphasizing potential fungal‐driven stability and adaptive microbial interactions in nutrient‐limited soils. Tight cross‐domain interactions in retrogressed soils enhance microbial network robustness, even as diversity and abundance within individual groups decline. Fungal taxa emerge as central stabilizers, with enriched potential for P mobilization and aromatic compound degradation, while N‐fixing genes, phototrophic protists, and DNA viruses may further support nutrient cycling and community dynamics. Ultimately, these findings underscore the importance of holistic, cross‐domain frameworks in understanding ecosystem stability and microbial adaptation under extreme nutrient limitation.

## MATERIALS AND METHODS

### Study sites and sampling

Study sites were located along the Ecological Staircase in Jug Handle State Natural Reserve near Fort Bragg, CA (39.377 N and 123.813 W). This chronosequence consists of five marine terraces, ranging from 100,000 to 500,000 years old, formed by ocean erosion and tectonic uplift. The terraces experience a Mediterranean climate with warm, dry summers and cool, wet winters^19^. The youngest terraces (100,000 and 200,000 years old, terrace 1 and terrace 2, respectively) support a productive mixed conifer forest dominated by Bishop pine (*Pinus muricata*) on fertile soils (Inceptisols), representing the buildup and peak of succession^23^. The oldest terraces (over 300,000 and 400,000 years old, terraces 3 and 4) are in the retrogressive stage of succession, with nutrient‐poor, acidic soils (Spodosols), and feature Pygmy Forests of stunted vegetation (<4 m tall after 100 years^22^). Previous studies in these terraces have shown that N and P speciation change significantly with soil age. For example, the mineral N pool (mainly NH_4_^+^) declines sharply from ~300 mg N/m^2^ at the youngest terrace to 0 mg N/m^2^ at the oldest terrace^21^. Total P declines steadily from the youngest to middle terraces, and then stabilizes. The proportion of labile and mineral P decreases with soil age, while organic and occluded P become more abundant^20^. Additionally, organic P has been shown to bind with iron (Fe) and aluminum (Al), making it less bioavailable^5^. We focused our analyses on terrace 1 (the young terrace), which represents the buildup stage of succession, and on terraces 3 (mid‐age) and 4 (old), which represent retrogressive stages. These terraces share key ectomycorrhizal hosts (*Pinus muricata* and *P. contorta*) and, in the older sites, a similar assemblage of ericoid shrubs (*Rhododendron macrophyllum*, *Vaccinium ovatum*, and *Gaultheria shallon*), providing a consistent biological framework for comparing microbial and biogeochemical patterns^27^. Terrace 2, by contrast, supports a transitional vegetation community where early‐successional conifers co‐occur with ericaceous shrubs^27^, introducing heterogeneity in plant composition and nutrient status. Excluding this intermediate terrace allowed us to focus on more evident ecosystem contrasts and capture representative stages of soil and vegetation development along the retrogression gradient. In each terrace, nine independent soil samples were collected at 3–4 m intervals, totaling 27 samples across terraces. The organic surface layer (O horizon) was carefully removed before sampling to ensure consistent collection of mineral soil. In the young terrace (Terrace 1, Inceptisol), samples measured 20 × 20 × 20 cm to encompass the A1 mineral layer (0–20 cm). In the mid‐age (Terrace 3, Spodosol) and old (Terrace 4, Ultisol) terraces, where mineral horizons are thinner and more compacted, samples measured 20 × 20 × 10 cm, corresponding to the A and E layers (0–10 cm). Sampling design followed established soil profiles from previous work on the Mendocino Ecological Staircase^29^, ^69^. Samples were homogenized by hand mixing, and 200 g of soil samples were air‐dried, sieved through a 2 mm sieve, pulverized, and used to measure pH, SOM by combustion, total P using Mehlich3‐ICP, TOC, and total N using elemental analysis (Table S1 and Figure S1). For DNA extraction, 25 g aliquots were placed into sterilized stainless‐steel grinding jars with grinding balls (Qiagen) and submerged in liquid nitrogen for 1 min. After snap‐freezing, samples were pulverized in a Tissue Lyser II (Qiagen) using two 5‐min cycles at 25 Hz, with 1‐min liquid nitrogen freezing between cycles.

### DNA extraction

Given the cross‐domain nature of our study, including protists, we extracted DNA from 5 g soil samples to maximize microbial diversity detection. Protist densities in soil range from 10³ to 10⁶ cells per gram^70^, meaning that extracting DNA from only 100–200 mg of soil, as traditionally done, could capture as few as 100–10,000 cells, potentially under‐representing their diversity. For DNA extraction, 5 g of pulverized soil was transferred to a 50‐ml Lysing Matrix E tube (MP Biomedicals) containing 15 ml of extraction buffer (5% CTAB, 0.5 M NaCl, 240 mM K_2_HPO_4_, pH 8.0) and 15 ml of phenol:chloroform:isoamyl alcohol (25:24:1). Samples were bead‐beaten (Fast Prep; MP Biomedicals) at 4 m/s for 30 s (twice) and then centrifuged at 7000*g* for 10 min. The supernatant was transferred to a clean tube with 15 ml of chloroform:isoamyl alcohol (24:1) and centrifuged at 7,000 *g* for 10 min at 4°C. DNA was precipitated using 15 ml of isopropanol and 10 μl of linear acrylamide (Ambion), incubated for 10 min at room temperature, and centrifuged at 10,000*g* for 10 min at 4°C. The pellet was washed twice with 70% ethanol, air‐dried, and dissolved in 300 μl of DEPC‐treated water. To further purify the DNA, 100 μl of crude extract was processed using magnetic beads (Speedbeads; GE Healthcare) in a solution containing 18% polyethylene glycol 8000, 1 M NaCl, 10 mM Tris‐HCl (pH 8), 1 mM EDTA (pH 8), and 0.05% Tween 20. Samples were incubated at 300 rpm for 10 min, placed on a magnetic stand for 5 min, and washed twice with 80% ethanol. Beads were air‐dried for 5 min, eluted with 100 μl of sterile DEPC‐treated water, incubated at 500 rpm for 5 min, and placed back on the magnetic stand. The final DNA extract was collected and the DNA concentration was measured using a Qubit fluorometer (Thermo Fisher Scientific). Extraction efficiency was determined by re‐extracting soil samples, yielding 90%–93% efficiency.

### Amplicon library preparation, sequencing, and analysis

Amplicon libraries were prepared using a two‐step barcoding approach described by Herbold et al.,^71^ with some modifications described in Ceja‐Navarro et al.^46^ Ribosomal sequences of the different microbial groups were amplified using the following primers: 515 F (5′‐GTGCCAGCMGCCGCGGTAA‐3′) and 806rb (5′‐GGACTACHVGGGTWTCTAAT‐3′) for prokaryotes^72^, ^73^, ITS1‐F (5′‐CTTGGTCATTTAGAGGAAGTAA‐3′) and ITS2‐R (5′‐GCTGCGTTCTTCATCGATGC‐3′) for fungi^74^ 1380 F (5′‐CCCTGCCHTTTGTACACAC‐3′), and 1510 R (5′‐CCTTCYGCAGGTTCACCTAC‐3′) for protists^75^. Prokaryotic and protist libraries were demultiplexed based on their unique barcodes and primers, and then trimmed to a uniform length. Sequences were processed as described in Ceja‐Navarro et al.^46^ Briefly, zero‐radius operational taxonomic units (ZOTUs) were inferred using unoise3 in USEARCH v11^76^. Representative sequences were screened against the NCBI nucleotide database using BLASTn (e‐value of 1e^–5^), and taxonomic filtering was carried out using MEGAN Community Edition v.6^77^. Filtered ZOTUs were taxonomically characterized using Sintax^76^ with a cutoff of 0.8 against the SILVA database (release 138.1)^78^ for prokaryotes and the PR2 database v.4.12.0^79^. Total sequences were mapped against corresponding ZOTUs at a 97% identity to account for PCR and sequencing errors, and to generate an abundance table. For phylogenetic analysis, ZOTUs were aligned using ClustalW^80^, and phylogenetic trees for each group were built with IQ‐TREE 2^81^ using model finder and ultrafast bootstrap approximation with 1000 replicates. Fungal sequences were processed using PIPITS v2.7^82^. Demultiplexed libraries were combined into a single fasta file and fungal ITS regions were extracted using the pipits_funits (‐x ITS1 option). Sequences were dereplicated, singletons were removed, and chimeras were filtered using the pipits_process. Input sequences were mapped to the chimera‐free representative sequences at 97% identity, and BIOM tables were generated. Representative sequences were classified with the RDP classifier against the UNITE fungal ITS reference dataset (v04.02.2020). A phylogenetic tree was reconstructed in QIIME2^83^ using the Ghostree plugin^84^ with the SILVA v123 and UNITE databases. Functional roles of fungi were defined using the FUNguild v1.0 database^85^, retaining only highly probable guilds. Protist feeding strategies were inferred for taxa identified at the genus level, based on published reports^86^, ^87^, ^88^, ^89^, ^90^, ^91^. A detailed table for protists' feeding preferences is available in Table S5.

The abundance table, mapping file, and phylogenetic tree were imported into R (v4.4.0) using the Phyloseq package^92^. For alpha diversity (Shannon index), libraries were subsampled to the minimum number of sequences among samples 100 times (seed number 3). Rarefaction analysis confirmed that this subsampling level represented the communities for the chosen diversity indices (Figure S12). Statistical significance of differences in alpha diversity was assessed using the Kruskal–Wallis test and Dunn's test with the Benjamini–Hochberg method for *p*‐value adjustment. A standardized multidiversity index was calculated from the observed richness and the Shannon index for all analyzed groups^45^. An analysis of the association between the multidiversity index based on observed richness and the one derived from the Shannon index showed a strong direct correlation (*r* = 0.91, *p* < 0.001). Based on this result, we used the Shannon index for all further analyses.

For beta‐diversity analysis, microbial abundance data were normalized using the variance–stabilization transformation in DESeq. 2^93^. Weighted Unifrac distance matrices were generated for each microbial group using the vegan package and ordinated with principal coordinate analysis (PCoA) in Phyloseq. Samples were categorized based on location, and variation in community composition was assessed using Adonis (nonparametric permutation multivariate analysis of variance) with 1000 permutations. Plant community composition (first PCoA axis) and diversity (Shannon index) were also quantified using 18S rRNA‐derived plant ZOTUs. To evaluate microbial compositional differences across terraces while accounting for soil and vegetation variables, the combined abundances of all microbial groups were transformed into a Bray–Curtis distance matrix and analyzed using a PERMANOVA test in vegan. Covariates included SOM, total N, TOC, total P, plant beta‐diversity, and plant Shannon index, and the PERMANOVA test was performed for the full and marginal models. To partition the variance into components uniquely attributable to terrace age, environmental covariates, and their shared effects, we used the *varpart* function in vegan. This analysis decomposed Bray–Curtis variation among samples into four components: the fraction explained only by environmental covariates, the fraction explained only by terrace age, the fraction jointly explained by both, and the unexplained residual fraction. Finally, associations between overall soil community composition and selected biotic and abiotic parameters were tested using Mantel correlations implemented in *ggcor* (v0.9.4.3).

### Quantification of microbial groups using qPCR

We used 10 ng of total DNA as a template for qPCR to quantify targeted soil organisms, using the primers described in the amplicon sequencing section. qPCR conditions were optimized with a temperature gradient, and amplification accuracy was confirmed by detecting a single band of the expected size. These bands were cloned, sequenced, and used as qPCR standards. qPCR reactions were performed in triplicate using a Bio‐Rad CFX96 Real‐Time PCR System. Each 25 μl reaction contained 1 μl of sample DNA or cloned standard, 12.5 μl of 2× iQ SYBR Green Supermix (Bio‐Rad), 0.4 μM of each primer, and 9.5 μl of nuclease‐free water. Thermal cycling conditions were as follows: initial denaturation at 95°C for 3 min; 35 cycles of 95°C for 10 s; and 55°C for 30 s, followed by a melt curve analysis to verify amplification specificity. Amplicon sizes were confirmed by gel electrophoresis. A linear relationship was observed between the log of the standard DNA copy number and the threshold cycle (*r* ^2^ = 0.98). PCR amplification efficiencies ranged from 98% to 105%. Runs outside this range (*r* ^2^ < 0.98 and efficiency <0.98 or >1.1) were repeated. Starting copy numbers for each sample were normalized per gram of soil and corrected by a factor of 0.9 for DNA extraction efficiency. Copy numbers were also adjusted based on the percentage of amplified sequences corresponding to each target group: 99.5% prokaryotic, 98.3% fungal, and 50.1% protists.

### Network reconstruction and analysis

Molecular ecological networks were reconstructed to investigate changes in community structure using random matrix theory (RMT)‐based co‐occurrence association network analysis. Networks were constructed individually for each analyzed soil group. Biom tables were rarefied in R and exported for network reconstruction. Networks were also reconstructed, combining all datasets. For these networks, raw counts for all groups were combined and then rarefied to the minimum number of sequences among all samples. Only ZOTUs detected in at least 6 of the 27 samples were used for network reconstruction. Network reconstruction was conducted with the molecular ecological network analyses pipeline (MENAP, http://ieg4.rccc.ou.edu/mena/) with the following settings: for missing data, fill blanks with 0.01 if data have paired values; normalize data using CLR; use the Spearman correlation similarity matrix; calculate by decreasing cutoff from the top; and for speed selection, regress Poisson distribution only. RMT was used to identify the appropriate correlation thresholds for network reconstruction^94^, ^95^. Indirect correlations were removed using iDirect^96^ to retain only robust, direct associations among microbial taxa. Various network topological indices were calculated in the MENAP interface to characterize the topological structure of all networks (Table S2)^32^. To test the significance of the constructed empirical networks, 100 random networks were generated following the Maslov–Sneppen procedure^97^. Network topological properties were recalculated with each randomization. Since network size and connectivity varied substantially among the different networks (Table S2), RM, which measures how modular a network is relative to the mean expected modularity^31^, was calculated as the ratio of the difference between the modularity of an empirical network and the mean modularity of randomized networks over that mean^32^.

### Network robustness and vulnerability

We analyzed network robustness and vulnerability in microbial networks containing 520 to 643 nodes, with varying connectivity and modularity. All analyses were conducted in R using the igraph package. Robustness, defined as the capacity of a network to maintain connectivity under node removal^98^, was assessed using (a) random removal, where nodes were selected randomly and (b) targeted removal, sequentially removing nodes based on degree centrality, starting with the highest‐degree nodes^32^. Each strategy was repeated across 1000 simulations per network to capture stochastic variation. At each removal step, the size of the largest connected component was recalculated using the igraph *components()* function as a measure of remaining network connectivity. Nodes were removed until the network was fully fragmented, and robustness was quantified as the area under the curve (AUC) of the largest component size across the removal sequence. Group‐specific robustness was assessed separately for predefined microbial groups (fungi and protists) using 100 iterations, following the same procedure to estimate their contributions to overall network connectivity.

Network vulnerability, defined as the reduction in global efficiency after node removal^32^, was evaluated for each network and microbial group. Nodes and their associated edges were removed using the igraph *delete_vertices()* function. For each network, maximum vulnerability and the mean and standard deviation of vulnerabilities were calculated, enabling comparisons of network vulnerability and the relative contribution of microbial groups (e.g., fungi and protists) toward maintaining global information flow. All code and datasets used for robustness and vulnerability analyses, including node removal procedures and calculations, are available at https://github.com/javiercnav/network_robustness_vulnerability.

### Cohesion

The Cohesion metric^47^, an abundance‐weighted, null model‐corrected metric based on pairwise correlations across taxa, was calculated as a measure of community complexity. Abundance tables were extracted from the original, unrarefied biom tables for the ZOTUS/OTUs retained in the reconstructed networks (the networked groups). Extracted abundance tables were used for Cohesion analysis using the CohesionScript_v1.1 from https://github.com/cherren8/Cohesion with a pers.cutoff of 0.3 and 1000 randomized iterations.

### Mechanisms of community assembly

The relative influences of community assembly processes were assessed using a phylogenetic bin‐based null model framework, iCAMP^99^. Detailed methods are described in Ceja‐Navarro et al.^46^.

### Metagenome sequencing and analysis

Metagenomic libraries were prepared using 500 ng of total DNA extracted from 5 g of soil per sample, with three replicates per terrace. DNA was quantified using a Qubit fluorometer, sheared to ~500 bp using a Diagenode Bioruptor sonicator, and assessed by agarose gel electrophoresis. Libraries were constructed using the NEBNext Ultra Library Prep Kit from NEB (E7370L) and sequenced on an Illumina NovaSeq instrument at 20 Gb per sample. Reads were curated with Trimmomatic and assembled using MEGAHIT^100^. Scaffolds across samples were dereplicated at 99% identity using PSI‐CD‐HIT to create a global assembly^101^. Reads were mapped to the scaffolds using Bowtie2 and Samtools^102^, ^103^. Gene models were predicted from the global assembly using Pyrodigal^104^ and annotated using the VEBA bioinformatics suite^105^ against UNIREF50/90^106^. Annotations of putative proteins of interest were validated against the NCBI nr database ver. 08‐06‐22 using DIAMOND^107^ and taxonomic classification confirmed in MEGAN Community Edition v.6^77^. Gene counts were determined using FeatureCounts and normalized in DEseq. 2 using variance‐stabilized normalization. Statistical significance of differences in gene counts was assessed using the Kruskal–Wallis test, followed by Dunn's test with the Benjamini–Hochberg correction. To explore the relationships between microbial functional potential and environmental variables, we examined correlations between normalized gene abundances and soil nutrient pools. Pearson correlations were calculated between each functional gene category and soil physicochemical parameters, including pH, total P, total N, TOC, and SOM. To account for covariation among these factors, we also computed partial correlations using a residual‐based approach: for each pair of variables, we first regressed both the gene abundance and the environmental variable against their relevant covariates (e.g., pH and SOM for total P; pH, OM, and total P for total N and TOC) and then correlated the resulting residuals. *p*‐values were adjusted for multiple testing using the Benjamini–Hochberg false discovery rate (FDR) correction. A piecewise structural equation model (piecewise SEM) was used to evaluate a parsimonious causal pathway linking soil chemistry to microbial functional potential via community composition. Soil total community composition was summarized as the first axis of a PCoA based on a Bray–Curtis distance matrix. Indices for selected genes were generated by *z*‐score scaling their normalized abundances. Linear models were then constructed for community composition as a function of soil pH, total P, and total N, and for each gene index as a function of community composition, pH, total P, and total N. Models were fitted using the piecewise SEM package in R (v4.4.0). We report standardized path coefficients (*β*), component model *R* ^2^ values, and Fisher's *C* as a global test of model fit. Due to the limited replication (*n* = 9), we did not perform AICc‐based model selection, as fitting multiple nested models would risk overparameterization. Instead, we used a parsimonious, hypothesis‐guided model structure constrained to the strongest and most ecologically relevant predictors (total N, total P, and pH).

Prokaryotic and micro‐eukaryotic MAGs were binned and characterized using VEBA v2.0. Briefly, prokaryotic bins were generated with Metabat2^108^ and CONCOCT^109^. CheckM2^110^ was used for quality assessment, retaining MAGs with a minimum completeness of 50% and a maximum contamination of 10%. MAG gene models were predicted with Pyrodigal^104^, and MAGs were classified using GTDB‐Tk^111^. Unclassified bins were then run through a consensus‐domain wrapper for Tiara, proteins were predicted with MetaEuk^112^, and MAG quality was assessed with BUSCO^113^. Poor‐quality MAGs (<25% completeness and >10% contamination) were removed, and the remaining were classified against eukaryote_odb10 in BUSCO. Unbinned scaffolds were screened for viral sequences using Virsorter2^114^ and CheckV^115^ following the standard operational procedures in https://www.protocols.io/view/viral-sequence-identification-sop-with-virsorter2-5qpvoyqebg4o/v3?step=3. Contigs >3 kb were analyzed using VirSorter2 targeting DNA viruses and the option “‐‐keep‐original‐seq,” and further processed with CheckV to trim host genes. Viral MAGs were dereplicated using 99% average nucleotide identity using PSI‐CD‐HIT. Gene modeling was performed using Prodigal‐GV and classification was carried out using VEBA against geNomad's taxonomy^116^.

Predicted genes and proteins from MAGs were annotated using VEBA against the Pfam^117^, Uniref50/90^106^, and KOFAMSCAN^118^. Proteins of interest were further annotated against the nr‐NCBI database using DIAMOND. AMGs involved in the P cycle were also verified using Phyre2^119^ with an alignment threshold >50% and a confidence of 100%. Viral MAG abundance was determined by summing gene counts per MAG, and the data were transformed into a Phyloseq object for analysis of diversity and community composition using a Bray–Curtis distance matrix.

Information regarding gene annotations and MAG taxonomies is available at: https://github.com/javiercnav/Supplementary_datasets_Ecological_Staircase.

Gene counts from all MAGs were determined using FeatureCounts, and genome abundance was calculated as the average variance‐stabilized counts normalized by the total gene count for each MAG. Statistical significance was assessed using a Kruskal–Wallis test with Dunn's test with the Benjamini–Hochberg correction. Relationships between diversity, community composition, functional group prevalence, predicted genes, cohesion, and biotic and abiotic environmental parameters were calculated using Pearson's correlation in R^120^.

### Phage–host prediction

Phage–host prediction was carried out using iPHoP (v1.2.0)^121^ against the default iPHoP host database and our generated MAGs using a minimum confidence score of 90%. Phage–host correlations for selected phage–host pairs were evaluated by Pearson correlation using normalized coverage values across all samples (“within‐sample”) and terrace‐averaged coverages (“cross‐sample”).

## AUTHOR CONTRIBUTIONS

**Javier A. Ceja‐Navarro**: Conceptualization; data curation; formal analysis; funding acquisition; investigation; methodology; project administration; resources; software; supervision; validation; visualization; writing—original draft; writing—review and editing. **Dishant Patel**: Data curation; investigation; writing—original draft; writing—review and editing. **Garret Genco**: data curation; investigation; writing—review and editing. **Alyssa Byer**: Investigation; methodology. **Daliang Ning**: Data curation. **Kenneth H. Wan**: Methodology. **Susan E. Celniker**: Funding acquisition; methodology; writing—review and editing. **Jizhong Zhou**: Supervision; writing—review and editing. **Paul Dijkstra**: Writing—review and editing. **Bruce A. Hungate**: Writing—review and editing. **Jennifer Pett‐Ridge**: Writing—review and editing. **Eoin L. Brodie**: Conceptualization; supervision; writing—review and editing.

## ETHICS STATEMENT

The authors have nothing to report.

## CONFLICT OF INTERESTS

The authors declare no conflict of interests.

## Supporting information

Supplementary_Tables.

Supplementary_text1.

Ceja‐Navarro_Supplementary_Figures_updated.

## Data Availability

Amplicon sequencing data are available at NCBI under BioProject accession number PRJNA1160002. Metagenomic sequencing data are available under the following SRA accession numbers, with corresponding BioSample accession numbers in parentheses: young terrace, SRR30413555 (SAMN38676622), SRR30413541 (SAMN43324171), and SRR30413538 (SAMN43324172); mid‐age terrace, SRR30413535 (SAMN38676623), SRR30413553 (SAMN43324173), and SRR30413551 (SAMN43324174); and old terrace, SRR30413548 (SAMN43324175), SRR30413545 (SAMN43324176), and SRR30413542 (SAMN43324177).
