# Supplementary material for: Ecosystem retrogression enhances cross‐domain microbial stability and increases the genetic potential for nutrient cycling
Source: mLife. 2026 Jul 19;5(4):486–506. doi: 10.1002/mlf2.70087 (PMC13529207; doi:10.1002/mlf2.70087)
Supplement: Supplementary file 1 [file MLF2-5-486-s001.pdf]

```
==== PIECEWISE SEM SUMMARY ====


Structural Equation Model of sem_mod

Call:
  PCoA1 ~ pH + total_P + TN
  bact_AP_index ~ PCoA1 + pH + total_P + TN
  fung_AP_index ~ PCoA1 + pH + total_P + TN
  fung_alkP_index ~ PCoA1 + pH + total_P + TN
  fung_phy_index ~ PCoA1 + pH + total_P + TN
  urease_index ~ PCoA1 + pH + total_P + TN
  urease_f_index ~ PCoA1 + pH + total_P + TN
  nif_index ~ PCoA1 + pH + total_P + TN

    AIC
 28.035

---
Tests of directed separation:

                          Independ.Claim Test.Type DF Crit.Value
P.Value
     fung_AP_index ~ bact_AP_index + ...      coef  3    -0.6345
0.5709
   fung_alkP_index ~ bact_AP_index + ...      coef  3    -0.6156
0.5817
    fung_phy_index ~ bact_AP_index + ...      coef  3    -0.2984
0.7849
      urease_index ~ bact_AP_index + ...      coef  3     0.5912
0.5960
    urease_f_index ~ bact_AP_index + ...      coef  3    -0.4175
0.7044
         nif_index ~ bact_AP_index + ...      coef  3    -0.7880
0.4882
   fung_alkP_index ~ fung_AP_index + ...      coef  3     0.2854
0.7939
    fung_phy_index ~ fung_AP_index + ...      coef  3     0.7175
0.5249
      urease_index ~ fung_AP_index + ...      coef  3     1.0595
0.3671
    urease_f_index ~ fung_AP_index + ...      coef  3     0.8764
0.4453
         nif_index ~ fung_AP_index + ...      coef  3     7.4032
0.0051 **
  fung_phy_index ~ fung_alkP_index + ...      coef  3     4.7446
0.0178  *
    urease_index ~ fung_alkP_index + ...      coef  3    -1.6509
0.1973
  urease_f_index ~ fung_alkP_index + ...      coef  3     3.9923
```

```
0.0282  *
       nif_index ~ fung_alkP_index + ...      coef  3     0.0396
0.9709
     urease_index ~ fung_phy_index + ...      coef  3    -0.7889
0.4877
   urease_f_index ~ fung_phy_index + ...      coef  3     6.1037
0.0088 **
        nif_index ~ fung_phy_index + ...      coef  3     0.3703
0.7358
     urease_f_index ~ urease_index + ...      coef  3    -0.7544
0.5054
          nif_index ~ urease_index + ...      coef  3     1.1718
0.3259
        nif_index ~ urease_f_index + ...      coef  3     0.5859
0.5991

--
Global goodness-of-fit:

Chi-Squared = Inf with P-value = 0 and on 18 degrees of freedom
Fisher's C = 56.436 with P-value = 0.067 and on 42 degrees of freedom

---
Coefficients:

         Response Predictor Estimate Std.Error DF Crit.Value P.Value
Std.Estimate
            PCoA1        pH  -0.1237    0.1227  5    -1.0084  0.3595
-0.3851
            PCoA1   total_P  -0.0173    0.0471  5    -0.3682  0.7278
-0.1289
            PCoA1        TN  -1.3467    0.5402  5    -2.4928  0.0550
-0.4979
    bact_AP_index     PCoA1  -3.4879    0.6391  4    -5.4578  0.0055
-1.0226 **
    bact_AP_index        pH  -0.5554    0.1924  4    -2.8870  0.0447
-0.5067  *
    bact_AP_index   total_P   0.1179    0.0682  4     1.7281  0.1590
0.2570
    bact_AP_index        TN   2.0098    1.1561  4     1.7383  0.1571
0.2179
    fung_AP_index     PCoA1   4.1996    1.3283  4     3.1616  0.0341
1.2312  *
    fung_AP_index        pH  -1.1320    0.3998  4    -2.8311  0.0473
-1.0328  *
    fung_AP_index   total_P   0.5071    0.1418  4     3.5761  0.0232
1.1052  *
    fung_AP_index        TN   2.4481    2.4030  4     1.0188  0.3659
0.2654
  fung_alkP_index     PCoA1   5.1708    1.3069  4     3.9566  0.0167
```

```
1.5160  *
  fung_alkP_index        pH  -0.6006    0.3934  4    -1.5268  0.2015
-0.5480
  fung_alkP_index   total_P   0.4572    0.1395  4     3.2768  0.0306
0.9964  *
  fung_alkP_index        TN   1.6528    2.3643  4     0.6991  0.5230
0.1792
   fung_phy_index     PCoA1   4.3118    2.2077  4     1.9531  0.1225
1.2641
   fung_phy_index        pH  -0.6196    0.6646  4    -0.9324  0.4039
-0.5654
   fung_phy_index   total_P   0.3111    0.2357  4     1.3198  0.2574
0.6779
   fung_phy_index        TN   2.2618    3.9940  4     0.5663  0.6015
0.2452
     urease_index     PCoA1  -3.6708    1.1083  4    -3.3122  0.0296
-1.0762  *
     urease_index        pH  -2.0545    0.3336  4    -6.1582  0.0035
-1.8745 **
     urease_index   total_P   0.4647    0.1183  4     3.9272  0.0171
1.0127  *
     urease_index        TN   6.0398    2.0050  4     3.0124  0.0395
0.6547  *
   urease_f_index     PCoA1   3.9231    1.6930  4     2.3172  0.0814
1.1502
   urease_f_index        pH  -1.0002    0.5096  4    -1.9626  0.1212
-0.9126
   urease_f_index   total_P   0.4056    0.1807  4     2.2443  0.0882
0.8841
   urease_f_index        TN   2.4813    3.0628  4     0.8101  0.4633
0.2690
        nif_index     PCoA1   1.0611    3.4036  4     0.3117  0.7708
0.3111
        nif_index        pH  -1.8016    1.0245  4    -1.7585  0.1535
-1.6438
        nif_index   total_P   0.0753    0.3634  4     0.2072  0.8460
0.1641
        nif_index        TN  10.7560    6.1575  4     1.7468  0.1556
1.1659

  Signif. codes:  0 '***' 0.001 '**' 0.01 '*' 0.05

---
Individual R-squared:

         Response method R.squared
            PCoA1   none      0.95
    bact_AP_index   none      0.99
    fung_AP_index   none      0.97
  fung_alkP_index   none      0.97
```

fung_phy_index	none	0.92
urease_index	none	0.98
urease_f_index	none	0.95
nif_index	none	0.82
