# Supplementary material for: Ecosystem retrogression enhances cross‐domain microbial stability and increases the genetic potential for nutrient cycling
Source: mLife. 2026 Jul 19;5(4):486–506. doi: 10.1002/mlf2.70087 (PMC13529207; doi:10.1002/mlf2.70087)
Supplement: Supplementary file 2 [file MLF2-5-486-s002.pdf]

Supplementary Table 1. Soil characterization data for three terraces along the ecological staircase chronosequence.

Terrace	Age (years)	Sampling depth	Total P (µg/g)	TOC (%)	Total N (%)	SOM (%)	Soil moisture (%)	pH	Dominant plant species*
Young	100,000	0-20 cm	5.00 ±0.80	6.03±0.60	0.27±0.02	9.8±0.95	24.8±0.10	5.8±0.10	*Pinus contorta, Pinus muricata, Pinus radiata*
Mid-age	300,000	0-10 cm	2.20 ±0.34	3.23±0.60	0.07±0.01	4.6±0.76	7.7±0.26	4.4±0.08	*Pinus contorta, Pinus muricata, Arctostaphylos nummularia,Rhododendron macrophyllum, Vaccinium ovatum, Gaultheria shallon*
old	400,000	0-10 cm	1.80±0.37	5.31±0.40	0.01±0.008	7.1±0.71	14.1±0.23	4.0±0.06	*Pinus contorta, Pinus muricata, Arctostaphylos nummularia,Rhododendron macrophyllum, Vaccinium ovatum, Gaultheria shallon*

Values are weighted means across horizons ± 1 SE. TOC – total organic carbon. * Based on the reports by Dynarski and Houlton, 2020.

Supplementary Table 2. Network topological properties for empirical and 100 randomized networks

Network Indexes	Terrace	Group	Empirical network	100 randomized networks (mean ± SD)
Average clustering coefficient (avgCC)	young	prokaryotes	0.27	0.005 ± 0.002
Average path distance (GD)	young	prokaryotes	12.899	5.292 ± 0.049
Geodesic efficiency (E)	young	prokaryotes	0.138	0.208 ± 0.002
Harmonic geodesic distance (HD)	young	prokaryotes	7.251	4.803 ± 0.035
Centralization of degree (CD)	young	prokaryotes	0.014	0.014 ± 0.000
Centralization of betweenness (CB)	young	prokaryotes	0.094	0.048 ± 0.007
Centralization of stress centrality (CS)	young	prokaryotes	2990.963	0.130 ± 0.022
Centralization of eigenvector centrality (CE)	young	prokaryotes	0.293	0.180 ± 0.029
Density (D)	young	prokaryotes	0.004	0.004 ± 0.000
Reciprocity	young	prokaryotes	1	1.000 ± 0.000
Transitivity (Trans)	young	prokaryotes	0.423	0.009 ± 0.002
Connectedness (Con)	young	prokaryotes	0.213	0.865 ± 0.022
Efficiency	young	prokaryotes	0.986	0.997 ± 0.000
Hierarchy	young	prokaryotes	0	0.000 ± 0.000
Lubness	young	prokaryotes	1	1.000 ± 0.000
Modularity(fast_greedy)	young	prokaryotes	0.868	0.619 ± 0.005
Average clustering coefficient (avgCC)	mid-age	prokaryotes	0.231	0.005 ± 0.003
Average path distance (GD)	mid-age	prokaryotes	6.642	6.838 ± 0.261
Geodesic efficiency (E)	mid-age	prokaryotes	0.25	0.175 ± 0.006
Harmonic geodesic distance (HD)	mid-age	prokaryotes	4.003	5.735 ± 0.187
Centralization of degree (CD)	mid-age	prokaryotes	0.015	0.015 ± 0.000
Centralization of betweenness (CB)	mid-age	prokaryotes	0.025	0.110 ± 0.023
Centralization of stress centrality (CS)	mid-age	prokaryotes	0.13	0.179 ± 0.039
Centralization of eigenvector centrality (CE)	mid-age	prokaryotes	0.436	0.270 ± 0.046
Density (D)	mid-age	prokaryotes	0.007	0.007 ± 0.000
Reciprocity	mid-age	prokaryotes	1	1.000 ± 0.000
Transitivity (Trans)	mid-age	prokaryotes	0.414	0.008 ± 0.006
Connectedness (Con)	mid-age	prokaryotes	0.096	0.712 ± 0.049
Efficiency	mid-age	prokaryotes	0.953	0.994 ± 0.000
Hierarchy	mid-age	prokaryotes	0	0.000 ± 0.000
Lubness	mid-age	prokaryotes	1	1.000 ± 0.000

Modularity(fast_greedy)	mid-age	prokaryotes	0.922	0.770 ± 0.010
Average clustering coefficient (avgCC)	old	prokaryotes	0.314	0.016 ± 0.004
Average path distance (GD)	old	prokaryotes	5.201	3.868 ± 0.027
Geodesic efficiency (E)	old	prokaryotes	0.256	0.285 ± 0.002
Harmonic geodesic distance (HD)	old	prokaryotes	3.911	3.507 ± 0.020
Centralization of degree (CD)	old	prokaryotes	0.032	0.032 ± 0.000
Centralization of betweenness (CB)	old	prokaryotes	0.133	0.045 ± 0.006
Centralization of stress centrality (CS)	old	prokaryotes	2.939	0.155 ± 0.018
Centralization of eigenvector centrality (CE)	old	prokaryotes	0.28	0.160 ± 0.016
Density (D)	old	prokaryotes	0.012	0.012 ± 0.000
Reciprocity	old	prokaryotes	1	1.000 ± 0.000
Transitivity (Trans)	old	prokaryotes	0.362	0.021 ± 0.003
Connectedness (Con)	old	prokaryotes	0.361	0.959 ± 0.018
Efficiency	old	prokaryotes	0.973	0.990 ± 0.000
Hierarchy	old	prokaryotes	0	0.000 ± 0.000
Lubness	old	prokaryotes	1	1.000 ± 0.000
Modularity(fast_greedy)	old	prokaryotes	0.715	0.434 ± 0.005
Average clustering coefficient (avgCC)	young	fungi	0.27	0.044 ± 0.009
Average path distance (GD)	young	fungi	4.033	3.318 ± 0.054
Geodesic efficiency (E)	young	fungi	0.33	0.342 ± 0.004
Harmonic geodesic distance (HD)	young	fungi	3.034	2.925 ± 0.034
Centralization of degree (CD)	young	fungi	0.062	0.062 ± 0.000
Centralization of betweenness (CB)	young	fungi	0.115	0.066 ± 0.009
Centralization of stress centrality (CS)	young	fungi	0.781	0.218 ± 0.027
Centralization of eigenvector centrality (CE)	young	fungi	0.269	0.164 ± 0.015
Density (D)	young	fungi	0.03	0.030 ± 0.000
Reciprocity	young	fungi	1	1.000 ± 0.000
Transitivity (Trans)	young	fungi	0.362	0.061 ± 0.008
Connectedness (Con)	young	fungi	0.424	0.959 ± 0.031
Efficiency	young	fungi	0.941	0.974 ± 0.001
Hierarchy	young	fungi	0	0.000 ± 0.000
Lubness	young	fungi	1	1.000 ± 0.000
Modularity(fast_greedy)	young	fungi	0.669	0.402 ± 0.008
Average clustering coefficient (avgCC)	mid-age	fungi	0.287	0.050 ± 0.008
Average path distance (GD)	mid-age	fungi	3.647	3.105 ± 0.043

Geodesic efficiency (E)	mid-age	fungi	0.364	0.366 ± 0.004
Harmonic geodesic distance (HD)	mid-age	fungi	2.75	2.732 ± 0.028
Centralization of degree (CD)	mid-age	fungi	0.065	0.065 ± 0.000
Centralization of betweenness (CB)	mid-age	fungi	0.112	0.055 ± 0.010
Centralization of stress centrality (CS)	mid-age	fungi	2.613	0.190 ± 0.029
Centralization of eigenvector centrality (CE)	mid-age	fungi	0.236	0.141 ± 0.014
Density (D)	mid-age	fungi	0.039	0.039 ± 0.000
Reciprocity	mid-age	fungi	1	1.000 ± 0.000
Transitivity (Trans)	mid-age	fungi	0.381	0.069 ± 0.007
Connectedness (Con)	mid-age	fungi	0.433	0.959 ± 0.027
Efficiency	mid-age	fungi	0.924	0.966 ± 0.001
Hierarchy	mid-age	fungi	0	0.000 ± 0.000
Lubness	mid-age	fungi	1	1.000 ± 0.000
Modularity(fast_greedy)	mid-age	fungi	0.651	0.365 ± 0.009
Average clustering coefficient (avgCC)	old	fungi	0.359	0.101 ± 0.013
Average path distance (GD)	old	fungi	4.452	2.817 ± 0.039
Geodesic efficiency (E)	old	fungi	0.333	0.405 ± 0.004
Harmonic geodesic distance (HD)	old	fungi	3.002	2.472 ± 0.023
Centralization of degree (CD)	old	fungi	0.116	0.116 ± 0.000
Centralization of betweenness (CB)	old	fungi	0.14	0.073 ± 0.011
Centralization of stress centrality (CS)	old	fungi	0.894	0.277 ± 0.030
Centralization of eigenvector centrality (CE)	old	fungi	0.218	0.174 ± 0.014
Density (D)	old	fungi	0.057	0.057 ± 0.000
Reciprocity	old	fungi	1	1.000 ± 0.000
Transitivity (Trans)	old	fungi	0.475	0.136 ± 0.010
Connectedness (Con)	old	fungi	0.619	0.978 ± 0.023
Efficiency	old	fungi	0.92	0.950 ± 0.001
Hierarchy	old	fungi	0	0.000 ± 0.000
Lubness	old	fungi	1	1.000 ± 0.000
Modularity(fast_greedy)	old	fungi	0.591	0.315 ± 0.009
Average clustering coefficient (avgCC)	young	protists	0.211	0.004 ± 0.002
Average path distance (GD)	young	protists	6.23	6.079 ± 0.097
Geodesic efficiency (E)	young	protists	0.222	0.186 ± 0.003
Harmonic geodesic distance (HD)	young	protists	4.514	5.379 ± 0.075
Centralization of degree (CD)	young	protists	0.013	0.013 ± 0.000

Centralization of betweenness (CB)	young	protists	0.024	0.067 ± 0.010
Centralization of stress centrality (CS)	young	protists	0.279	0.141 ± 0.022
Centralization of eigenvector centrality (CE)	young	protists	0.209	0.215 ± 0.033
Density (D)	young	protists	0.004	0.004 ± 0.000
Reciprocity	young	protists	1	1.000 ± 0.000
Transitivity (Trans)	young	protists	0.334	0.008 ± 0.003
Connectedness (Con)	young	protists	0.115	0.753 ± 0.031
Efficiency	young	protists	0.975	0.996 ± 0.000
Hierarchy	young	protists	0	0.000 ± 0.000
Lubness	young	protists	1	1.000 ± 0.000
Modularity(fast_greedy)	young	protists	0.875	0.726 ± 0.006
Average clustering coefficient (avgCC)	mid-age	protists	0.188	0.011 ± 0.004
Average path distance (GD)	mid-age	protists	5.562	4.589 ± 0.064
Geodesic efficiency (E)	mid-age	protists	0.256	0.245 ± 0.003
Harmonic geodesic distance (HD)	mid-age	protists	3.905	4.075 ± 0.049
Centralization of degree (CD)	mid-age	protists	0.03	0.030 ± 0.000
Centralization of betweenness (CB)	mid-age	protists	0.086	0.075 ± 0.014
Centralization of stress centrality (CS)	mid-age	protists	0.315	0.193 ± 0.035
Centralization of eigenvector centrality (CE)	mid-age	protists	0.304	0.228 ± 0.025
Density (D)	mid-age	protists	0.01	0.010 ± 0.000
Reciprocity	mid-age	protists	1	1.000 ± 0.000
Transitivity (Trans)	mid-age	protists	0.319	0.019 ± 0.005
Connectedness (Con)	mid-age	protists	0.234	0.869 ± 0.035
Efficiency	mid-age	protists	0.969	0.992 ± 0.000
Hierarchy	mid-age	protists	0	0.000 ± 0.000
Lubness	mid-age	protists	1	1.000 ± 0.000
Modularity(fast_greedy)	mid-age	protists	0.764	0.578 ± 0.007
Average clustering coefficient (avgCC)	old	protists	0.27	0.038 ± 0.006
Average path distance (GD)	old	protists	6.006	3.381 ± 0.035
Geodesic efficiency (E)	old	protists	0.242	0.330 ± 0.002
Harmonic geodesic distance (HD)	old	protists	4.132	3.033 ± 0.023
Centralization of degree (CD)	old	protists	0.061	0.061 ± 0.000
Centralization of betweenness (CB)	old	protists	0.245	0.053 ± 0.007
Centralization of stress centrality (CS)	old	protists	2.105	0.220 ± 0.027
Centralization of eigenvector centrality (CE)	old	protists	0.248	0.159 ± 0.017

Density (D)	old	protists	0.021	0.021 ± 0.000
Reciprocity	old	protists	1	1.000 ± 0.000
Transitivity (Trans)	old	protists	0.368	0.054 ± 0.005
Connectedness (Con)	old	protists	0.644	0.947 ± 0.023
Efficiency	old	protists	0.973	0.982 ± 0.000
Hierarchy	old	protists	0	0.000 ± 0.000
Lubness	old	protists	1	1.000 ± 0.000
Modularity(fast_greedy)	old	protists	0.66	0.380 ± 0.006
Average clustering coefficient (avgCC)	young	combined	0.317	0.011 ± 0.003
Average path distance (GD)	young	combined	8.676	4.251 ± 0.035
Geodesic efficiency (E)	young	combined	0.188	0.258 ± 0.002
Harmonic geodesic distance (HD)	young	combined	5.317	3.881 ± 0.023
Centralization of degree (CD)	young	combined	0.027	0.027 ± 0.000
Centralization of betweenness (CB)	young	combined	0.074	0.052 ± 0.006
Centralization of stress centrality (CS)	young	combined	120.547	0.188 ± 0.022
Centralization of eigenvector centrality (CE)	young	combined	0.254	0.184 ± 0.021
Density (D)	young	combined	0.007	0.007 ± 0.000
Reciprocity	young	combined	1	1.000 ± 0.000
Transitivity (Trans)	young	combined	0.398	0.017 ± 0.002
Connectedness (Con)	young	combined	0.296	0.950 ± 0.017
Efficiency	young	combined	0.982	0.994 ± 0.000
Hierarchy	young	combined	0	0.000 ± 0.000
Lubness	young	combined	1	1.000 ± 0.000
Modularity(fast_greedy)	young	combined	0.782	0.477 ± 0.004
Average clustering coefficient (avgCC)	mid-age	combined	0.341	0.016 ± 0.003
Average path distance (GD)	mid-age	combined	4.456	3.627 ± 0.022
Geodesic efficiency (E)	mid-age	combined	0.287	0.301 ± 0.001
Harmonic geodesic distance (HD)	mid-age	combined	3.484	3.319 ± 0.015
Centralization of degree (CD)	mid-age	combined	0.025	0.025 ± 0.000
Centralization of betweenness (CB)	mid-age	combined	0.012	0.025 ± 0.003
Centralization of stress centrality (CS)	mid-age	combined	2.155	0.101 ± 0.010
Centralization of eigenvector centrality (CE)	mid-age	combined	0.194	0.107 ± 0.010
Density (D)	mid-age	combined	0.012	0.012 ± 0.000
Reciprocity	mid-age	combined	1	1.000 ± 0.000
Transitivity (Trans)	mid-age	combined	0.384	0.022 ± 0.003

Connectedness (Con)	mid-age	combined	0.259	0.963 ± 0.013
Efficiency	mid-age	combined	0.961	0.990 ± 0.000
Hierarchy	mid-age	combined	0	0.000 ± 0.000
Lubness	mid-age	combined	1	1.000 ± 0.000
Modularity(fast_greedy)	mid-age	combined	0.728	0.377 ± 0.005
Average clustering coefficient (avgCC)	old	combined	0.375	0.030 ± 0.003
Average path distance (GD)	old	combined	3.416	3.159 ± 0.016
Geodesic efficiency (E)	old	combined	0.368	0.347 ± 0.001
Harmonic geodesic distance (HD)	old	combined	2.714	2.884 ± 0.010
Centralization of degree (CD)	old	combined	0.038	0.038 ± 0.000
Centralization of betweenness (CB)	old	combined	0.01	0.023 ± 0.003
Centralization of stress centrality (CS)	old	combined	0.124	0.126 ± 0.013
Centralization of eigenvector centrality (CE)	old	combined	0.205	0.102 ± 0.007
Density (D)	old	combined	0.018	0.018 ± 0.000
Reciprocity	old	combined	1	1.000 ± 0.000
Transitivity (Trans)	old	combined	0.427	0.039 ± 0.002
Connectedness (Con)	old	combined	0.266	0.981 ± 0.012
Efficiency	old	combined	0.939	0.984 ± 0.000
Hierarchy	old	combined	0	0.000 ± 0.000
Lubness	old	combined	1	1.000 ± 0.000
Modularity(fast_greedy)	old	combined	0.654	0.280 ± 0.004

Supplementary Table 3. Network vulnerability calculated on the networks for the combined datasets

Terrace	Group	Maximum Vulnerability	Mean Vulnerability	Standard deviation
Young	Combined	0.093	0.0017	0.007
Mid-age	Combined	0.012	0.0006	0.003
Old	Combined	0.019	0.0007	0.003

Supplementary Table 4. Pearson and partial correlations between normalized functional gene abundances and soil physicochemical parameters along the retrogression gradient.

Gene_Index	Nutrient	Pearson_*r*	Pearson_*p*	Partial_*r*	Partial_*p*	Controls	FDR_Pearson	FDR_Partial
cellulases_bacteria	pH	-0.975	7.73E-06	-0.869	0.00236	total_P, org_mat	0.0006	0.1185
acid_P_bacteria	TN	0.962	3.24E-05	0.354	0.349	pH, org_mat, total_P	0.0010	0.4653
alkaline_P_bacteria	TN	0.959	4.34E-05	0.605	0.0844	pH, org_mat, total_P	0.0010	0.2150
amoA	TN	0.919	0.000467	0.396	0.292	pH, org_mat, total_P	0.0052	0.4291
cellulases_bacteria	total_P	-0.916	0.000531	0.367	0.331	pH, org_mat	0.0052	0.4652
cellulases_fungi	pH	-0.91	0.000651	-0.758	0.0181	total_P, org_mat	0.0052	0.1185
cellulases_fungi	TN	-0.913	0.000584	-0.651	0.0575	pH, org_mat, total_P	0.0052	0.1739
laccase_fungi	pH	-0.917	0.000496	-0.65	0.0578	total_P, org_mat	0.0052	0.1739
urease_fungi	pH	-0.914	0.000557	-0.774	0.0143	total_P, org_mat	0.0052	0.1185
acid_P_fungi	pH	-0.902	0.000889	-0.821	0.00663	total_P, org_mat	0.0059	0.1185
alkaline_P_bacteria	pH	0.899	0.000985	0.316	0.407	total_P, org_mat	0.0059	0.4967
alkaline_P_bacteria	total_P	0.9	0.000958	0.364	0.336	pH, org_mat	0.0059	0.4652
phytase_fungi	pH	-0.894	0.00115	-0.65	0.058	total_P, org_mat	0.0059	0.1739
bacteria_xylanases	pH	-0.894	0.00115	-0.65	0.058	total_P, org_mat	0.0059	0.1739
urease_bacteria	TN	0.891	0.00127	0.767	0.0159	pH, org_mat, total_P	0.0061	0.1185
alkaline_P_fungi	TN	-0.886	0.00146	-0.672	0.0472	pH, org_mat, total_P	0.0064	0.1739
amoA	total_P	0.883	0.00158	0.327	0.391	pH, org_mat	0.0064	0.4891
laccase_fungi	TN	-0.883	0.00159	-0.828	0.00583	pH, org_mat, total_P	0.0064	0.1185

amoA	pH	0.878	0.00186	0.206	0.594	total_P, org_mat	0.0070	0.6289
phytase_fungi	TN	-0.869	0.00238	-0.789	0.0115	pH, org_mat, total_P	0.0080	0.1185
alkaline_P_fungi	pH	-0.867	0.00251	-0.706	0.0334	total_P, org_mat	0.0080	0.1603
bacteria_xylanases	TN	-0.869	0.00238	-0.789	0.0115	pH, org_mat, total_P	0.0080	0.1185
urease_fungi	TN	-0.866	0.00254	-0.674	0.0464	pH, org_mat, total_P	0.0080	0.1739
acid_P_fungi	TN	-0.863	0.00272	-0.385	0.306	pH, org_mat, total_P	0.0082	0.4406
laccase_fungi	total_P	-0.858	0.00307	0.231	0.549	pH, org_mat	0.0088	0.6081
laccase_bacteria	TN	0.856	0.00324	0.512	0.158	pH, org_mat, total_P	0.0090	0.3164
laccase_bacteria	total_P	0.851	0.00364	0.602	0.0866	pH, org_mat	0.0097	0.2150
acid_P_bacteria	pH	0.838	0.00484	0.358	0.344	total_P, org_mat	0.0124	0.4653
cellulases_fungi	total_P	-0.831	0.00546	0.458	0.216	pH, org_mat	0.0136	0.3616
phytase_fungi	total_P	-0.824	0.00627	0.302	0.43	pH, org_mat	0.0137	0.4994
acid_P_bacteria	total_P	0.829	0.00575	0.209	0.589	pH, org_mat	0.0137	0.6289
bacteria_xylanases	total_P	-0.824	0.00627	0.302	0.43	pH, org_mat	0.0137	0.4994
urease_fungi	total_P	-0.827	0.006	0.489	0.181	pH, org_mat	0.0137	0.3342
cellulases_bacteria	TN	-0.808	0.00839	0.652	0.0571	pH, org_mat, total_P	0.0178	0.1739
acid_P_fungi	total_P	-0.799	0.00977	0.615	0.0781	pH, org_mat	0.0200	0.2083
xylanases_fungi	pH	-0.797	0.01	-0.522	0.15	total_P, org_mat	0.0200	0.3164
laccase_bacteria	pH	0.786	0.012	-0.305	0.425	total_P, org_mat	0.0234	0.4994
polyphenol_ox_fungi	pH	-0.781	0.0131	-0.844	0.00421	total_P, org_mat	0.0248	0.1185
alkaline_P_fungi	total_P	-0.778	0.0135	0.457	0.216	pH, org_mat	0.0249	0.3616
urease_bacteria	total_P	0.761	0.0171	0.643	0.0616	pH, org_mat	0.0308	0.1739

nitrogenase	pH	-0.758	0.018	-0.56	0.117	total_P, org_mat	0.0316	0.2705
xylanases_fungi	total_P	-0.732	0.0251	0.207	0.594	pH, org_mat	0.0430	0.6289
nitrogenase	total_P	-0.711	0.0318	0.084	0.831	pH, org_mat	0.0532	0.8427
urease_bacteria	pH	0.692	0.039	-0.448	0.226	total_P, org_mat	0.0638	0.3616
urease_bacteria	TOC	0.651	0.0574	-0.285	0.457	pH, org_mat, total_P	0.0918	0.5223
acid_P_bacteria	TOC	0.645	0.0609	-0.507	0.163	pH, org_mat, total_P	0.0933	0.3164
pmoA	pH	-0.646	0.06	-0.725	0.0272	total_P, org_mat	0.0933	0.1506
polyphenol_ox_fungi	total_P	-0.637	0.065	0.706	0.0334	pH, org_mat	0.0975	0.1603
xylanases_fungi	TN	-0.6	0.0879	0.418	0.262	pH, org_mat, total_P	0.1266	0.3930
polyphenol_ox_bacteria	TN	0.602	0.0862	0.554	0.122	pH, org_mat, total_P	0.1266	0.2705
polyphenol_ox_bacteria	TOC	0.555	0.121	0.002	0.995	pH, org_mat, total_P	0.1708	0.9950
pmoA	total_P	-0.549	0.126	0.454	0.219	pH, org_mat	0.1745	0.3616
polyphenol_ox_fungi	TN	-0.53	0.142	0.51	0.161	pH, org_mat, total_P	0.1929	0.3164
alkaline_P_bacteria	TOC	0.513	0.158	-0.642	0.0625	pH, org_mat, total_P	0.2107	0.1739
amoA	TOC	0.463	0.209	-0.552	0.124	pH, org_mat, total_P	0.2736	0.2705
nitrogenase	TN	-0.43	0.248	0.332	0.383	pH, org_mat, total_P	0.3189	0.4891
cellulases_fungi	TOC	-0.4	0.286	0.503	0.167	pH, org_mat, total_P	0.3550	0.3164
laccase_bacteria	TOC	0.401	0.285	-0.769	0.0154	pH, org_mat, total_P	0.3550	0.1185
pmoA	TOC	0.392	0.297	0.643	0.0615	pH, org_mat, total_P	0.3624	0.1739
alkaline_P_fungi	TOC	-0.382	0.31	0.466	0.207	pH, org_mat, total_P	0.3718	0.3616
polyphenol_ox_bacteria	total_P	0.379	0.315	0.153	0.694	pH, org_mat	0.3718	0.7242

polyphenol_ox_bacteria	pH	0.351	0.354	-0.115	0.768	total_P, org_mat	0.4111	0.7899
acid_P_fungi	TOC	-0.319	0.404	0.759	0.0176	pH, org_mat, total_P	0.4601	0.1185
laccase_fungi	TOC	-0.315	0.409	0.28	0.466	pH, org_mat, total_P	0.4601	0.5243
phytase_fungi	TOC	-0.305	0.425	0.325	0.394	pH, org_mat, total_P	0.4636	0.4891
bacteria_xylanases	TOC	-0.305	0.425	0.325	0.394	pH, org_mat, total_P	0.4636	0.4891
urease_fungi	TOC	-0.298	0.437	0.448	0.226	pH, org_mat, total_P	0.4696	0.3616
pmoA	TN	-0.277	0.47	0.432	0.245	pH, org_mat, total_P	0.4976	0.3753
nitrogenase	TOC	0.265	0.492	0.641	0.0628	pH, org_mat, total_P	0.5134	0.1739
cellulases_bacteria	TOC	-0.224	0.562	0.574	0.106	pH, org_mat, total_P	0.5781	0.2544
polyphenol_ox_fungi	TOC	0.077	0.844	0.744	0.0216	pH, org_mat, total_P	0.8559	0.1296
xylanases_fungi	TOC	-0.059	0.88	0.432	0.245	pH, org_mat, total_P	0.8800	0.3753

Pairwise (Pearson) and partial correlation coefficients (r) were calculated between variance-stabilized gene abundances and key soil variables (pH, total P, total N, total organic carbon, and organic matter). Partial correlations controlled for intercorrelated soil covariates as specified under the “Controls” column. False discovery rate (FDR)–adjusted p-values are provided for both Pearson and partial tests. Gene indices represent normalized abundances of bacterial and fungal functional genes related to phosphorus (phosphatases, phytases), nitrogen (ureases, nitrogenase), and carbon cycling (cellulases, xylanases, laccases, and polyphenol oxidases).

Supplementary Table 5. Protist genera, feeding preferences, and morphology

Genus	Feeding preference	Morphology	Author	DOI
Acanthamoeba	omnivore	naked amoeba	Rodriguez Zaragoza	10.1007/s00248-003-1056-1
Actinosphaerium	omnivore, predator	hypotrichous ciliate	Lawler, 1993	10.1007/BF00317669
Adelina	entomogenous parasite	coccidia (single-celled intracellular parasite)	Lange & Wittenstein, 2001; Alfazairy et. al., 2020	10.1006/jipa.2000.499; 10.1186/s41938-020-0203-z
Adriamonas	bacterivore	naked flagellate	Verhagen et al, 1994	10.1016/S0932-4739(11)80076-3
Allantion	bacterivore	naked flagellate	Howe et al, 2009	10.1016/j.protis.2008.11.007
Allapsa	bacterivore	naked flagellate	Howe et al, 2009	10.1016/j.protis.2008.11.007
Allas	bacterivore	testate cell silica	Scoble & Cavalier Smith 2014	10.1016/j.ejop.2013.12.005
Amastigomonas	bacterivore	flagellate	Adl et al. 2019; Archibald, 2017	10.1111/jeu.12691; 10.1007/978-3-319-32669-6
Amoeba	bacterivore	amoeba (naked, lobose)	Rodriguez Zaragoza; Wilkinson et. al., 2012	10.1007/s00248-003-1056-1; 10.4467/16890027AP.12.015.0761
Anisonema	eukaryvore	flagellate	Archibald et. al., 2017; Larsen & Patterson, 1990	10.1007/978-3-319-32669-6; 10.1080/00222939000770571
Anoplophrya	Osmotrophic	ciliate	Adl, 2018; Fernandez-Galiano & Perez-Uz, 1990	10.1111/jeu.12691; 10.1016/S0003-9365(11)80150-8
Anteholosticha	omnivore (bacteria, small protists like diatoms)	ciliate	Jung et. al., 2016; Feng et. al., 2010	10.4467/16890027AP.16.008.4943; 10.1007/s13131-010-0067-2
Aphanomyces	facultative parasite; saprotrophs or parasites	fungi	Archibald, 2017; Westcott & Horst, 2008	10.1007/978-3-319-32669-6; 10.1007/978-1-4020-4585-1_748
Apusomonas	bacterivore	flagellate	Adl, 2018; Lee, 2015	10.1111/jeu.12691; 10.4467/16890027AP.15.005.2192
Arachnula	bacterivore	naked amoeboid	Adl, 2018	10.1111/jeu.12691
Arcuospathidium_1	omnivore (ciliates, flagellates, starch grains)	ciliate	Foissner et. al., 2014; Foissner & Xu, 2007	10.4467/16890027AP.14.015.1596; 10.1007/1-4020-4735-5_5
Ascogregarina	Parasite (invertebrates, mainly mosquitos)	gregarine, small sporozoite, trophozoite attaches via epimerite	Pereira et. al., 2018	10.5007/2175-7925.2018v31n3p1

Aspidisca	omnivore	ciliate	Adl, 2018; Valbonesi, 1996	10.1111/jeu.12691; 10.1080/11250009609356162
Assulina	omnivore	testate amoeboflagellate/ amoeba (silica)	Meisterfeld 2000	https://protistologists.org/publications/illustrated-guide-to-the-protozoa/
Asterochloris	phototroph; lichen photobiont	green algae	Armaleo et. al., 2019; Skaloud et. al., 2015	10.1186/s12864-019-5629-x; 10.1111/jpy.12295
Balamuthia	parasite (invades human/animal CNS; possibly feed on small amoebae when free-living stage, however only observed in vitro)	amoeba (flattened, elongate); only trophozoite and cyst life stages	Visvesvara et. al., 2007; Archibald, 2017	10.1111/j.1574-695X.2007.00232.x; 10.1007/978-3-319-32669-6
Bicosoeca	bacterivore	flagellate	Tong et. al., 1998	10.1016/S0932-4739(98)80027-8
Bicosoecida	bacterivore	flagellate	Archibald, 2017	10.1007/978-3-319-32669-6
Blepharisma	bacterivore	ciliate	Adl, 2018; Giese & Smith, 1973	10.1111/jeu.12691; 10.2307/4444465
Bodo	bacterivore	flagellate	Archibald et. al., 2017; Turley et. al., 1988	10.1007/978-3-319-32669-6 ; 10.1016/0198-0149(88)90001-5
Bodomorpha	bacterivore	naked flagellate	Howe et al, 2009	10.1016/j.protis.2008.11.007
Botrydiopsis	phototroph (chlorophyll a only)	algae (possess flagellum)	McLeod, 1961; Archibald, 2017	10.1104/pp.36.1.114 ; 10.1007/978-3-319-32669-6
Bracteacoccus	phototroph	green algae (coccoid)	Kuikova et. al., 2012	10.1127/0029-5035/2012/0067
Bresslaua	carnivore	ciliate	Claff et. al., 2017; Coleman et. al., 2000	10.2307/1537789; 10.1079/9780851993942.0000
Bryometopus	Predator	ciliate	Adl, 2018	10.1111/jeu.12691
Bryophrya	omnivore	ciliate	Adl, 2018	10.1111/jeu.12691
Capsellina	omnivore	testate amoeboflagellate/ amoeba/flagellate (organic/agglutinated)	Howe et al, 2011	10.1016/j.protis.2010.10.002
Cavernomonas	bacterivore	naked flagellate	Bass et al, 2009	10.1016/j.protis.2009.01.004
Cercomonas	omnivore	naked amoeboflagellate	Ekelund, 1998	10.1016/S0038-0717(97)00266-6
Chaos	bacterivore/cytotrophic	naked amoeba	Adl, 2018	10.1111/jeu.12691
Chlamydomonas	phototroph	green algae, flagellated (apical)	Harris, 1989	10.1016/c2009-0-02778-0
Chlorococcum	phototroph (autotroph)	microalgae	Cabanelas et. al., 2017; Cabije et. al., 2009	10.1016/j.algal.2017.08.010; 10.1002/apj.329
Chloroidium	phototroph (able to grow heterotrophically)	green algae (coccoid)	Nelson et. al., 2017; Darienko et. al., 2010	10.7554/eLife.25783; 10.1080/09670260903362820

Chlorosarcina	phototroph	green algae (unicellular, coccoid, able to form cellular aggregates)	Bolivar-Galiano et. al., 2020	10.3390/app10186603
Coccomyxa	phototroph (lichen photobiont, ciliate symbiont, or free-living)	green algae (unicellular)	Verma et. al., 2009; Malavasi et. al., 2016	10.1111/j.1529-8817.2009.00718.x; 10.1371/journal.pone.0151137
Cochliopodium	Yeast, bacterivore	amoebae	Rodriguez Zaragoza; Sawyer, 1971	10.1007/s00248-003-1056-1 ; 10.2307/3224896
Coenocystis	phototroph	green algae	Hanagata & Chihara, 1999	https://www.jstage.jst.go.jp/article/jjapbot/74/4/74_74_4_9356/_pdf/-char/en
Colloderma	bacterivore	slime mold (brown/black spores in mass)	Leontyev et. al., 2019	10.11646/phytotaxa.399.3.5
Colpoda	omnivore	ciliate	Adl, 2018; Lynn & Malcolm, 2011	10.1111/jeu.12691; 10.1139/z83-041
Colpodella	predator (protists, algae)	biflagellate (small, free-living, forms cyst/withdraws flagella after feeding)	Archibald, 2017	10.1007/978-3-319-32669-6
Cryptodifflugia	bacterivore, large taxa predators (nematodes, 2 taxa)	testate amoeba	Geisen et. al., 2015; Bobrov & Mazei, 2017	10.1111/1462-2920.12949; 10.11646/zootaxa.4282.2.4
Cryptosporidium1	parasite	amoeba	Archibald et. al., 2017; Sente et. al., 2016	10.1007/978-3-319-32669-6 ; 10.1186/s40249-016-0162-5
Cultellothrix	predator (rotifers)	ciliate	Foissner, 2003	http://www.wfoissner.at/data_prot/Foissner_2003_47-54ap.pdf
Cyrtohymena	omnivore	ciliate	Adl, 2018; Kim et. al., 2012	10.1111/jeu.12691; 10.12651/JSR.2012.1.1.078
Cyrtolophosis	omnivore (small bacteria/picocyanobacteria)	ciliate (smaller)	Adl, 2018; Lynn, 2008	10.1111/jeu.12691; 10.1007/978-1-4020-8239-9
Dermamoeba	bacterivore (also ingest cyanobacteria)	amoeba (flattened, elongate)	Adl, 2018; Archibald, 2017	10.1111/jeu.12691; 10.1007/978-3-319-32669-6
Dictyochloropsis	phototroph (sometimes as photobiont)	green algae (coccal, reticulate chloroplast); free-living and lichenized algae	Taylor & Francis, 2007 (publishers); Dal Grande et. al., 2014	10.1080/09670260701440166; 10.1111/nph.12678
Didymium	bacterivore	slime mold	Clark et. al., 2002	10.2307/3761861
Dileptus	predator	ciliate	Adl, 2018; Vdacny et. al., 2011	10.1111/jeu.12691; 10.1016/j.ejop.2011.04.006
Dimastigella	bacterivore	flagellate	Archibald et. al., 2017; Vickerman, 1978	10.1007/978-3-319-32669-6; 10.2307/3226165
Diplophrys	heterotrophic	unicellular, colorless, test made of overlapping scales	Anderson & Cavalier-Smith, 2012; Archibald, 2017	10.4467/16890027AP.12.023.0783; 10.1007/978-3-319-32669-6

Echinamoeba	bacterivore	amoeba (free-living, tubular)	Rodriguez Zaragoza; Adl, 2018	10.1007/s00248-003-1056-1; 10.1111/jeu.12691; 10.1016
Elliptochloris	phototroph	green algae	Darienko et. al., 2016	10.1111/jpy.12481
Endostelium	bacterivore	amoeba	Olive et. al., 1984	10.2307/3793145
Eocercomonas	omnivore	naked amoeboflagellate	Bass et al, 2009; Ekelund 1998	10.1016/j.protis.2009.01.004; 10.1016/S0038-0717(97)00266-6
Euglena	photoautotrophic	flagellate	Archibald et. al., 2017	10.1007/978-3-319-32669-6
Euglypha	omnivore	testate amoeboflagellate/ amoeba (silica)	Meisterfeld 2000	https://protistologists.org/publications/illustrated-guide-to-the-protozoa/
Euplotes	omnivore	ciliate	Adl, 2018; Kusch, 1993	10.1111/jeu.12691; 10.1007/BF00566974
Eustigmatos	phototroph	microalgae (coccoid, unicellular, single flagellum in flagellated stage)	Kryvenda et. al., 2018	10.1080/09670262.2018.1475015
Exocolpoda	microphagous	ciliate	Foissner et. al., 2002	http://www.wfoissner.at/data_prot/Foissner_etal_2002_1-1063.pdf
Filamoeba	Bacterivore	amoeba	Rodriguez Zaragoza; Page, 1967	10.1007/s00248-003-1056-1; 10.2307/3224262
Flectomonas	bacterivore	naked flagellate	Howe et al, 2009	10.1016/j.protis.2008.11.007
Geminigera	mixotrophic (phototroph, bacterivore)	nanoflagellate	McKie-Krisberg et. al., 2015	10.1007/s00248-014-0543-x
Glaeseria	bacterivore	naked amoeba	Zhang & Leuders, 2017; Adl, 2018	10.1093/femsec/fix103; 10.1111/jeu.12691
Gloeocystis	photoautotrophic	green algae	Maxwell & Neuman, 1994; Kim, 2014	10.1016/0038-0717(94)90162-7; 10.5141/ecoenv.2014.038
Goniomonas	bacterivore	zooflagellate	Archibald, 2017; von der Heyden et. al., 2004	10.1007/978-3-319-32669-6; 10.1080/09670260400005567
Gonostomum	omnivore	ciliate (hypotrich)	Adl, 2018; Berger, 1999	10.1111/jeu.12691; 10.1007/978-94-011-4637-1
Gregarina1	parasite	cephaline gregarine, trophozoite stage, globular or cylindrical papilla-like epimerite	Archibald, 2017; Kundu & Haldar, 1981	10.1007/978-3-319-32669-6; 10.1007/bf00930500
Hafniomonas	phototroph (chloroplasts)	green algae (flagellated)	Hanus Ettl et. al., 1980	10.1007/bf00983186
Halteria	omnivore; bacterivore	ciliate	Adl, 2018; Simek et. al., 2000	10.1111/jeu.12691; 10.3354/ame022043

Haptoglossa	parasite (endoparasitic)	fungi	Archibald, 2017; Glockling & Beakes, 2000	10.1007/978-3-319-32669-6; 10.1017/S0953756299008916
Hartmannella	Bacterivore	amoeba	Rodriguez Zaragoza; Sleigh, 1991	10.1007/s00248-003-1056-1; Sleigh
Hedriocystis	bacterivore	testate cell (organic)	Bass et. al., 2009a	10.1016/j.protis.2008.07.002
Heleopera	mixotrophic (some heterotrophic)	testate amoeba	Adl, 2018; Mitchell & Gilbert, 2005	10.1111/jeu.12691; 10.1111/j.1550-7408.2004.tb00400.x
Hemichloris	photoautotrophic	green algae (2 chloroplasts)	Obbels et. al., 2016; Tschermak-Woess et. al., 2006	10.1093/femsec/fiw041; 10.1007/s00606-005-0382-x
Hemiophrys	carnivore (ciliates)	ciliate	Fenchel, 1968	10.1080/00785326.1968.10409626
Heterochlamydomonas	photoautotrophic	unicellular algae, motile vegetative cells with flagella	Samolov et. al., 2020; Langford & Cox, 1971	10.3390/microorganisms8071047; Langford
Heterophrys	omnivore (bacteria, small flagellates)	small heliozoa, fine organic spicules	Bardele, 1975	10.1007/bf00222116
Hyphochytrium	range from saprotrophic to parasitic	fungi	Dick, 2013	10.1007/978-94-015-9733-3
Korotnevella	eukaryvores and bacterivores (for smaller sized sp.)	amoeba (nonmineralized scales)	O'Kelley et. al., 2001	10.1111/j.1550-7408.2001.tb00205.x
Lacrymaria	Predator (unicellular organisms)	ciliate (spindle-shaped)	Adl, 2018; Mast, 1969	10.1111/jeu.12691; 10.1037/h0073607
Lamproderma	bacterivore	slime mold (channeled stipes, large sporangia, well-developed capillitium)	Abdel-Azeem & Fatma, 2013; Kowalski, 1968	10.5943/mycosphere/4/4/15; 10.2307/3757381
Leidyana1	parasite	gregarine, globular epimerite, trophozoite stage	Clopton, 1995	10.2307/3226836
Leptomyxa	Predator; omnivores; Protozoa-eaters and bacterivores	naked amoeba	Rodriguez Zaragoza; Adle, 2018; Cortes-Perez et. al., 2014	10.1007/s00248-003-1056-1; 10.1111/jeu.12691; 10.1007/s00248-013-0305-1
Leptopharynx	bacterivore	ciliate	Adl, 2018; Bamforth, 1995	10.1111/jeu.12691; 10.1007/978-94-011-0479-1_16
Limnofila	bacterivore	naked amoeboflagellate	Howe et al, 2011	10.1016/j.protis.2010.10.002
Litonotus	Predator	ciliate	Adl, 2018; Verni, 1985	10.1111/jeu.12691; 10.1007/bf00312065
Loxophyllum	Predator	ciliate	Mironova et. al., 2012	10.1093/plankt/fbr095
Maryna	bacterivore	ciliate	Lynn, 2010	10.1007/978-1-4020-8239-9_17

Mattesia	parasite (insects)	neogregarine, sporozoites develop into spindle-shaped trophozoites	Yaman & Radek, 2015	10.1007/s00436-015-4497-9
Mayorella	Protozoa and Algae; mostly carnivore	amoeba	Rodriguez Zaragoza; Glotova et. al., 2018	10.1007/s00248-003-1056-1; 10.4467/16890027AP.18.002.8396
Mesofila	bacterivore	naked amoeboflagellate	Bass et al, 2009	10.1016/j.protis.2009.01.004
Metabolomonas	bacterivore	naked amoeboflagellate	Brabender, 2012	10.1016/j.protis.2012.02.002
Metaurostylopsis	omnivore (bacteria, flagellates, small ciliates)	ciliate	Chen et. al., 2011; Borong et. al., 2016	10.1111/j.1463-6409.2010.00451.x; 10.1007/s11802-016-3148-9
Microdiaphanosoma	bacterivore	ciliate	Lynn, 2008; Quintela-Alonso et. al., 2011	10.1007/978-1-4020-8239-9; 10.1111/j.1550-7408.2010.00527.x
Micronuclearia	bacterivore (type species)	naked filose amoeba, branching/thread-like pseudopodia	Mikrjukov & Kylnikov, 2001	10.1078/0932-4739-00783
Monocystis	parasite (mainly earthworms)	sporocyst-forming (biconical shape); non-septate, motile sporonts, eugegarine	Velavan et. al., 2009; Kamm, 1922	10.1017/S0031182009990722; 10.2307/3221671
Monosiga	bacterivore	choanoflagellate	Boenigk & Arndt, 2000	10.3354/ame022243
Myrmecia	phototroph (lichen photobiont)	green algae (spherical, lobed chloroplast)	Honegger, 2009; Flechtner et. al., 2013	10.1007/978-3-540-87407-2_16; 10.3398/042.006.0103
Nannochloropsis	phototroph (able to grow mixotrophically, Xu)	microalgae (non-motile, spherical cells)	Zanella & Vianello, 2020; Xu et. al., 2004	10.1016/j.jff.2020.103919; 10.1023/B:BILE.0000045626.38354.1a
Nebela	carnivore	testate amoeba (silica)	Adl, 2018; Simpson & Volcani, 2012	10.1111/jeu.12691; 10.1007/978-1-4612-5944-2
Neobodo	bacterivore	flagellate	Archibald, 2017; Tikhonenkov et. al., 2016	10.1007/978-3-319-32669-6; :10.1111/jeu.12271
Nucleocercomonas	bacterivore	naked amoeboflagellate	Brabender, 2012	10.1016/j.protis.2012.02.002
Nudifila	Bacterivore	naked amoeboflagellate	Howe et al, 2011	10.1016/j.protis.2010.10.002
Ochromonas	mixotrophic, can grow as photoheterotrophs	flagellate	Zhang et. al., 2018	10.1016/j.scitotenv.2018.05.196
Orthamphisiella	bacterivore	ciliate	Thompson et. al., 2019; Garcia-Moyano et. al., 2015	10.1017/S0954102019000361; 10.3390/microorganisms3040667
Orthoamphisiella	omnivore	ciliate	Eigner et al. 1993	https://doi.org/10.1016/S0003-9365(11)80330-1

Oxnerella	algivore	naked amoeba	Dobell, 1917; Cavalier-Smith & Chao, 2012	10.1016/j.protis.2011.12.005
Oxytricha	omnivore	ciliate	Adl, 2018; Rae & Spear, 1978	10.1111/jeu.12691; 10.1073/pnas.75.10.4992
Paracercomonas	Bacterivore	naked amoeboflagellate	Bass et al, 2009	10.1016/j.protis.2009.01.004
Parachloroidium	phototrophic	microalgae (unicellular, coccoid)	Neustupa et. al., 2013	10.2216/13-142.2
Paraflabellula	bacterivore	amoebae (frequently adopt flabellate form; similar to Flabellula, fan-shaped)	Cortes-Perez et. al., 2014; Smirnov et. al., 2016	10.1007/s00248-013-0305-1; 10.1016/j.protis.2016.10.006
Paraphysomonas	bacterivore	flagellate (siliceous spine)	Archibald, 2017	10.1007/978-3-319-32669-6
Paraschneideria	parasite (insects)	septate gregarine; trophozoite with simple epimerite	Simdyanov et. al. 2017, Adl, 2018	10.7717/peerj.3354; 10.1111/jeu.12691
Parietochloris	photoautotrophic	green microalgae	Tababa et. al., 2012; Shibzukhova et. al., 2017	10.1007/s10811-011-9709-9; 10.3103/S0096392517030099
Paulinella	omnivore	testate amoeboflagellate/ amoeba (silica)	Meisterfeld 2000	https://protistologists.org/publications/illustrated-guide-to-the-protozoa/
Peregrinia	bacterivore	naked amoeboflagellate	Howe et al, 2011	10.1016/j.protis.2010.10.002
Perkinsiella-like	endosymbiont (obligate symbionts of paramoebid amoebae)	amoebae (P. amoebae/PLOs); closest relative kinetoplastid flagellate	Dykova et. al., 2003; Archibald, 2017	10.1078/0932-4739-00901; 10.1007/978-3-319-32669-6
Phalansterium	bacterivore	flagellate	Patterson, 1999	10.1086/303287
Phyllosiphon	endophytic parasite	green algae	Prochazkova et. al., 2016	10.11646/phytotaxa.283.2.3
Placocista	mixotrophic (some exclusively phagotrophic--bacteria?)	testate amoeba	Lara & Gomaa, 2017; Gomaa et. al., 2014	10.1142/9781786340580_0013; 10.1016/j.protis.2014.01.002
Platyophrya	cytrophic (ingests protists for growth, in addition to bacteria that are co-ingested)	ciliate	Adl, 2018; Foissner & Foissner, 1995	10.1111/jeu.12691; 10.1016/s0932-4739(11)80088-x
Platyreta	eukaryvore	naked amoeba	Bass et. al., 2009a	10.1016/j.protis.2008.07.002
Poterioochromonas	mixotrophic (cyanobacteria, green alga, diatoms, chrysomonads)	golden algae	Zhang et. al., 2009	10.1016/j.watres.2009.04.003
Proleptomonas	bacterivore	naked flagellate	Vickerman et al 2002	10.1078/1434-4610-00079
Prorocentrum	phototroph	dinoflagellate	Nimer et. al., 1999; Archibald, 2017	10.1104/pp.120.1.105; 10.1007/978-3-319-32669-6

Protacanthamoeba	bacterivore	amoeba (lobose; acanthopodia present)	Delafont et. al., 2014; Smirnov, 2009	10.1021/es5036255; 10.1016/B978-012373944-5.00359-X
Protaspa	eukaryvore	testate amoeboflagellate/ amoeba/flagellate (organic/agglutinated)	Howe et al, 2011	10.1016/j.protis.2010.10.002
Protostelium	omnivore (most prefer yeast, some bacteria or coccoid green algae)	sporocarpic amoebae	Adl, 2018	10.1111/jeu.12691
Pseudocyrtolophosis	bacterivore	ciliate	terHorst, 2010	10.1086/657047
Pseudodifflugia	eukaryvore	testate cell (organic)	Howe et. al., 2011; Wylezich et. al., 2002	10.1016/j.protis.2010.10.002; 10.1111/j.1550-7408.2002.tb00352.x
Pseudomonocystis	parasite	spherical gametocyst, biconical spore	Desportes & Schrevel, 2013	10.1163/9789004256057
Pseudoplatyophrya	fungivore (yeast, small fungi)	ciliate	Alekperov et. al., 2017; Adl, 2000	10.21685/1680-0826-2017-11-2-1; 10.1079/9780851996615.0001
Pseudouroleptus	omnivore (cyanobacteria, naked amoebae, flagellates, diatoms, testate amoebae, other ciliates)	ciliate	Berger, 2008	10.1007/978-1-4020-8917-6
Pseudourostyla	omnivore	ciliate	Adl, 2018; Berger, 2007	10.1111/jeu.12691; 10.1007/1-4020-5273-1_2
Ptolemeba	bacterivore	amoeba (limax-shaped, type species naked amoeba)	Watson et. al., 2014	10.1111/jeu.12139
Pyrsonympha	phagocytosis of wood (symbiosis with termite hindgut)	flagellate	Archibald, 2017; Bloodgood, 1975	10.1007/978-3-319-32669-6; 10.1016/0022-1910(75)90032-3
Pythium	plant pathogen (facultatively parasitic); can infect mammals; some saprotrophic	water mold (oomycete, fungus-like)	Archibald, 2017; McLeod et. al., 2009	10.1007/978-3-319-32669-6; 10.1016/j.mycres.2009.04.009
Reclinomonas	bacterivore	flagellate	O'Kelley, 1993	10.1111/j.1550-7408.1993.tb06120.x
Rhynchobodo	omnivore	flagellate	Lee et. al., 2003; Prokina & Mylnikov, 2017	10.1080/00785236.2003.10409506; 10.1134/S1995082917020110
Rhynchomonas	bacterivore	small flagellate	Archibald, 2017	10.1007/978-3-319-32669-6
Rigifila	bacterivore	filose; filopodia to draw in bacteria	Yabuki et. al., 2013	10.1016/j.protis.2012.04.005
Sandmanniella	omnivore (protists, bacteria)	ciliate	Adl, 2018; Foissner & Stoeck, 2009	10.1111/jeu.12691; 10.1111/j.1550-7408.2009.00429.x
Sandona	bacterivore	naked flagellate	Howe et al, 2009	10.1016/j.protis.2008.11.007
Schizoplasmodiopsis	bacterivore	amoeba (range from uninucleate, often branched amoeba to multinucleate plasmodia)	Olive & Stoianovitch, 1975	10.2307/3758827

Sphaeroeca	bacterivore	flagellate	Patterson & Hedley, 1996	10.1201/9781840765847
Sphenoderia	omnivore	testate amoeboflagellate/ amoeba (silica)	Meisterfeld 2000	https://protistologists.org/publications/illustrated-guide-to-the-protozoa/
Spirostomum	bacterivore	ciliate	Adl, 2018; Fokin et. al., 2005	10.1111/jeu.12691; 10.1007/s00709-004-0078-y
Spongomonas	Bacterivore	naked flagellate	Howe et al, 2011	10.1016/j.protis.2010.10.002
Spongospora	plant parariste	flagellate (unicellular, intracellular)	Neuhauser et al, 2014	10.1186/1471-2148-14-33
Spumella	bacterivore	flagellate	Bohme et. al., 2009; Archibald, 2017	10.1111/j.1574-6941.2009.00710.x; 10.1007/978-3-319-32669-6
Stenamoeba	bacterivore	naked amoeba	Adl, 2018; Geisen et. al., 2014	10.1111/jeu.12691; 10.1016/j.ejop.2014.01.007
Sterkiella	eukaryvore (bacteria, small amoeba, flagellates)	ciliate	Bharti et. al., 2018	10.1371/journal.pone.0207688
Stichococcus	phototroph	green microalgae (rod-shaped)	Ladislav et. al., 2016	10.1093/femsec/fiw122
Stichotrichia	bacterivore (and other small organisms/eukaryvore; occassionally feed on other organisms such as flagellates)	ciliate	Froud, 1949	https://doi.org/10.1242/jcs.s3-90.10.141
Tetracystis	phototroph	algae (biflagellate zoospores)	Remias et. al., 2010; Watanabe & Lewis, 2017	10.1007/s11099-010-0033-4; 10.2216/16-64.1
Tetramitus	bacterivore	amoeboflagellate (diverse morphology: amoeba, ciliated amoeba or amoeboid without cillium)	Archibald, 2017; Adl, 2018; Murtagh et. al., 2002	10.1078/0932-4739-00836Get ; 10.1007/978-3-319-32669-6; 10.1111/jeu.12691
Thaumatomonas	bacterivore	testate amoeboflagellate/ amoeba (silica)	Howe et al, 2011	10.1016/j.protis.2010.10.002
Tracheleuglypha	bacterivore	testate amoeba	Odgen & Couteaux, 1987; Adl, 2018	10.1016/S0932-4739(87)80005-6; 10.1111/jeu.12691
Trachelocorythion	omnivore	testate amoeboflagellate/ amoeba (silica)	Meisterfeld 2000	https://protistologists.org/publications/illustrated-guide-to-the-protozoa/
Trentepohlia	phototroph	algae (often appear orange/yellow/red, unique flagellar apparatus)	Uyenco, 1965; Brooks et. al., 2015	10.2307/3224534; 10.1094/PDIS-01-15-0029-FE
Trichonympha	wood (lignocellulose-digestng hindgut community of lower termites/cockroaches)	flagellate (multiflagellate)	James et. al., 2013; Archibald, 2017	10.1099/ijs.0.054874-0; 10.1007/978-3-319-32669-6
Trinema	omnivore	testate cell (silica)	Meisterfeld 2000	https://protistologists.org/publications/illustrated-guide-to-the-protozoa/
Urostyla	omnivore, cytrophic	ciliate	Adl, 2018; Turner, 1939	10.1111/jeu.12691; 10.2307/3222781

Vampyrella	eukaryvore	naked amoeba	Hess et. al. 2012	10.1371/journal.pone.0031165
Vannella	Bacterivore	small amoeba	Rodriguez Zaragoza; Bovee, 1965	10.1007/s00248-003-1056-1; 10.2307/3224288
Vexillifera	Bacterivore; may also feed on larger prey such as diatoms/algae/flagellates/ciliates/nematodes	large amoeba	Rodriguez Zaragoza; Munson, 1992	10.1007/s00248-003-1056-1; 10.2307/3226710
Viridiella	phototroph	algae (chlorella-like)	Albertano et. al., 1991	10.2216/i0031-8884-30-4-346.1
