# Supplementary material for: Ecosystem retrogression enhances cross‐domain microbial stability and increases the genetic potential for nutrient cycling
Source: mLife. 2026 Jul 19;5(4):486–506. doi: 10.1002/mlf2.70087 (PMC13529207; doi:10.1002/mlf2.70087)
Supplement: Supplementary file 3 [file MLF2-5-486-s003.docx]

**Supplementary Figures**

Supplementary Figure 1. Analyzed soil properties per terrace. N = 9 per terrace age. Significant differences were determined using ANOVA followed by Tukey’s HSD test.

Supplementary Figure 2. Variance partitioning of microbial community composition showing fractions of variation explained by environmental covariates [a], shared variation [c], terrace age [b], and unexplained variance [d]. Terrace age independently explained 13 % of the variance, environmental covariates 2 %, and 72 % was shared between both sets of predictors.

*Supplementary Figure 3. Relationships between total community composition (Bray–Curtis) and environmental variables. Edge width represents Mantel test r-values; edge color indicates significance. Pairwise Pearson correlations are shown using a color gradient, with square size indicating correlation strength. Sample size per terrace, n = 9.*

*Supplementary Figure 4. Network robustness, measured as the normalized Area Under the Curve (AUC) under random node removal strategies across microbial groups and terraces. Significant differences were calculated using Kruskal-Wallis followed by Dunn’s test with Benjamini-Hochberg p-value correction.*

Supplementary Figure 5. Cohesion changes for prokaryotes, fungi, and protists. Significant differences were assessed using ANOVA followed by Tukey’s HSD test. Sample size per terrace, n = 9.

Supplementary Figure 6. Changes in the abundances of selected prokaryotic populations identified as Metagenome-Assembled Genomes (MAGs) and their genetic potential. A) MAG normalized abundance across terraces. Circles are colored based on prokaryotic phylum. B) Presence/absence patterns of selected putative genes coding for proteins involved in P, C, and N cycling.

*Supplementary Figure 7. Correlations between functional gene abundances and soil physicochemical parameters across the retrogression gradient. (A) Pearson correlation coefficients (r) between variance-stabilized functional gene abundances and key soil variables (pH, total phosphorus [total P], total nitrogen [TN], and total organic carbon [TOC]). (B) Partial correlation coefficients controlling for covariation among soil variables. Colors indicate the direction and magnitude of the correlations (red = positive; blue = negative). Asterisks denote significance levels (p < 0.05 = *, p < 0.01 = **, p < 0.001 = ***). Gene categories represent normalized abundances of bacterial and fungal genes associated with carbon (cellulases, xylanases, laccases, polyphenol oxidases), nitrogen (ureases, methane and ammonia monooxygenases, nitrogenase), and phosphorus cycling (acid and alkaline phosphatases, phytases).*

*Supplementary Figure 8. Parsimonious piecewise Structural Equation Model (SEM) linking soil chemistry, microbial community composition, and functional gene potential. Arrows represent significant standardized path coefficients (β, p ≤ 0.10). Arrow color indicates effect direction (blue = positive, red = negative), and arrow thickness scales with –log₁₀(p), reflecting statistical support for each path. Node colors denote variable classes: soil parameters (brown), microbial community composition (yellow), bacterial functional gene indices (green), and fungal functional gene indices (magenta). Model R² values for endogenous variables are reported in the main text. The model explained most of the variance in community composition (R² = 0.95) and functional gene indices (R² = 0.82–0.99). Given the limited replication (n = 9), this SEM is presented as a parsimonious, hypothesis-generating framework emphasizing effect sizes and directional relationships rather than statistical significance.*

*Supplementary Figure 9. Significant Pearson correlations between prokaryotic gene abundance and positive cohesion.*

Supplementary Figure 10. Phylogenetic reconstruction for the PhoD protein predicted for phage Caudoviricetes.10952585 and reference sequences from the NCBI. Sequences were aligned using MAFFT, and the tree reconstruced with IQ-TREE using model finder and ultrafast bootstrap approximation with 1000 replicates. Labels in the tree branches represent the percent bootstrap support for the nodes.

*Supplementary Figure 11. Changes in soil phosphorus fractions across the Ecological Staircase chronosequence (adapted from Izquierdo et al., 2013, Biogeochemistry 367(1–2):135–47). The figure shows the relative contributions of mineral, labile, occluded, and organic P pools to total soil phosphorus across terraces spanning 100–500 ka. With increasing soil age, total P declines sharply, driven by the loss of labile and mineral forms and the accumulation of occluded and organic P. This shift provides the geochemical context for the observed enrichment of fungal phosphatase and phytase genes in older terraces.*

Supplementary Figure 12. Rarefaction curves for prokaryote, fungi, and protist sequences from soil collected from 3 marine terraces at the Ecological Staircase, Mendocino, California. Curves were generated up to the minimum sampling level for each group. N = 9 per terrace age.
